# A mu–delta opioid receptor brain atlas reveals neuronal co-occurrence in subcortical networks

**DOI:** 10.1007/s00429-014-0717-9

**Published:** 2014-03-13

**Authors:** Eric Erbs, Lauren Faget, Gregory Scherrer, Audrey Matifas, Dominique Filliol, Jean-Luc Vonesch, Marc Koch, Pascal Kessler, Didier Hentsch, Marie-Christine Birling, Manoussos Koutsourakis, Laurent Vasseur, Pierre Veinante, Brigitte L. Kieffer, Dominique Massotte

**Affiliations:** 1 Department of Neurogenetics and Translational Medicine, Institut de Génétique et de Biologie Moléculaire et Cellulaire, CNRS, INSERM, Université de Strasbourg, 1 rue Laurent Fries, BP10142, 67404 Illkirch cedex, France; 2 Department of Anesthesiology, Perioperative and Pain Medicine, Stanford Institute for Neuro-Innovation and Translational Neurosciences, Stanford University, Stanford, 94305 CA USA; 3 Imaging Centre, Institut de Génétique et de Biologie Moléculaire et Cellulaire, CNRS, INSERM, Université de Strasbourg, BP 10142, 1 rue Laurent Fries, 67404 Illkirch cedex, France; 4 Institut Clinique de la Souris, 1 rue Laurent Fries, 67404 Illkirch cedex, France; 5 Institut des Neurosciences Cellulaires et Intégratives CNRS UPR 3212, 5 rue Blaise Pascal, 67084 Strasbourg cedex 03, France; 6 Present Address: University of California, La Jolla, CA 92093 USA; 7 Present Address: Sanger Institute, Hinxton, Cambridge CB 10 1SA UK

**Keywords:** Mouse mu opioid receptor, Mouse delta opioid receptor, Receptor brain atlas, Heteromer, Aversive stimuli

## Abstract

Opioid receptors are G protein-coupled receptors (GPCRs) that modulate brain function at all levels of neural integration, including autonomic, sensory, emotional and cognitive processing. Mu (MOR) and delta (DOR) opioid receptors functionally interact in vivo, but whether interactions occur at circuitry, cellular or molecular levels remains unsolved. To challenge the hypothesis of MOR/DOR heteromerization in the brain, we generated redMOR/greenDOR double knock-in mice and report dual receptor mapping throughout the nervous system. Data are organized as an interactive database offering an opioid receptor atlas with concomitant MOR/DOR visualization at subcellular resolution, accessible online. We also provide co-immunoprecipitation-based evidence for receptor heteromerization in these mice. In the forebrain, MOR and DOR are mainly detected in separate neurons, suggesting system-level interactions in high-order processing. In contrast, neuronal co-localization is detected in subcortical networks essential for survival involved in eating and sexual behaviors or perception and response to aversive stimuli. In addition, potential MOR/DOR intracellular interactions within the nociceptive pathway offer novel therapeutic perspectives.

**Electronic supplementary material:**

The online version of this article (doi:10.1007/s00429-014-0717-9) contains supplementary material, which is available to authorized users.

## Introduction

Opioid receptors and endogenous opioid peptides are largely expressed throughout the nervous system (Le Merrer et al. 2009). The opioid system plays a key role in reward and motivation, and regulates emotional responses and cognition. The system also modulates nociception, neuroendocrine physiology and autonomic functions (Feng et al. 2012). The three opioid receptors mu (MOR), delta (DOR) and kappa (KOR) are homologous G protein-coupled receptors (GPCRs) (Filizola and Devi 2013), and their respective implication in pain control, drug abuse and mood disorders has been extensively studied (Pradhan et al. 2011; Lutz and Kieffer 2012).

Several decades of opioid pharmacology have uncovered the complexity of opioid system physiology. In particular, the analysis of opioid drug effects in vivo has revealed functional interactions across receptors, particularly documented for MORs and DORs. The best-known example is the implication of DORs in the development of analgesic tolerance to morphine, a prototypical MOR agonist (Cahill et al. 2007). Whether in vivo receptor interactions occur at systems level across neural circuits, within neurons via signaling pathways, or at molecular level by direct receptor–receptor contact, however, is highly debated, and is extremely difficult to tackle with existing tools.

Within a cell, functional interactions between two receptors may arise from a competition for downstream effectors or a shared association with intracellular partners within protein complexes. Intracellularly, the two receptors may also interact physically, and operate as homo- or heteromers with signaling and trafficking properties distinct from monomeric receptors (Rozenfeld and Devi 2011). The latter hypothesis stems from initial reports using recombinant cell systems in which receptor heteromerization has been demonstrated for many GPCRs including MOR and DOR (Jordan and Devi 1999; George et al. 2000). Whether these mechanisms indeed operate in vivo remains a central question in GPCR research. So far, little evidence supports in vivo MOR/DOR co-expression. In vivo co-localization has only been reported in dorsal root ganglia (DRG) (Wang et al. 2010; Scherrer et al. 2009; Rau et al. 2005), spinal cord (Gomes et al. 2004) and rostroventral medulla (Pedersen et al. 2011; Kivell et al. 2004). MOR/DOR co-expression was also reported in a limited number of brain areas using antibodies specifically raised against MOR–DOR heteromers (Gupta et al. 2010). As for most GPCRs, however, in-depth anatomical mapping of opioid receptors in the brain with subcellular resolution is still lacking.

The present study provides a proof-of-principle brain atlas for GPCR co-expression in vivo, using MOR and DOR as model interacting receptors. We previously targeted the enhanced Green Fluorescent Protein into the DOR gene locus to produce DOR-eGFP knock-in mice (Scherrer et al. 2006). Mutant animals express a fully functional receptor with a fused C-terminal eGFP (DOR-eGFP) in place of the native receptor and show no detectable alteration of behavior and responses to drugs. DOR-eGFP mice allowed visualizing DOR in vivo, with subcellular resolution, in DRGs (Scherrer et al. 2009), enteric neurons (Poole et al. 2011) and the hippocampus (Erbs et al. 2012). These mice were also instrumental to examine receptor trafficking in vivo upon drug treatment (Scherrer et al. 2006) or physiological challenge (Faget et al. 2012), and to understand implications for tolerance (Pradhan et al. 2009, 2011). Using a similar strategy, we have generated a second knock-in mouse line expressing MOR fused to the red fluorescent mcherry protein (MOR-mcherry). Breeding these animals with DOR-eGFP mice produced a bicolor double mutant line (DOR-eGFP/MOR-mcherry) that expresses functional fluorescent forms of the two receptors.

Here, we report fine mapping of MOR and DOR in nervous tissues with subcellular resolution. Fluorescent images corresponding to coronal and sagittal sections of the brain were collected and assembled to create a virtual atlas that can be freely searched at http://mordor.ics-mci.fr/. This is the first reported GPCR brain atlas, and the genetic approach is applicable to any GPCR/GPCR or GPCR/effectors pair. In addition, co-immunoprecipitation experiments uncovered mu–delta physical proximity in the hippocampus validating our approach to challenge the relevance of in vivo GPCR heteromers.

In-depth analysis of MOR and DOR distribution revealed that the two receptors are co-expressed in neurons from brain networks related to water and food consumption, sexual behavior or perception and responses to aversive stimuli that may endanger the animal. Localization in these key networks leads us to postulate that MOR/DOR neuronal co-expression is present in circuits essential for species survival.

## Materials and methods

### Animals

**Fig. 1:**
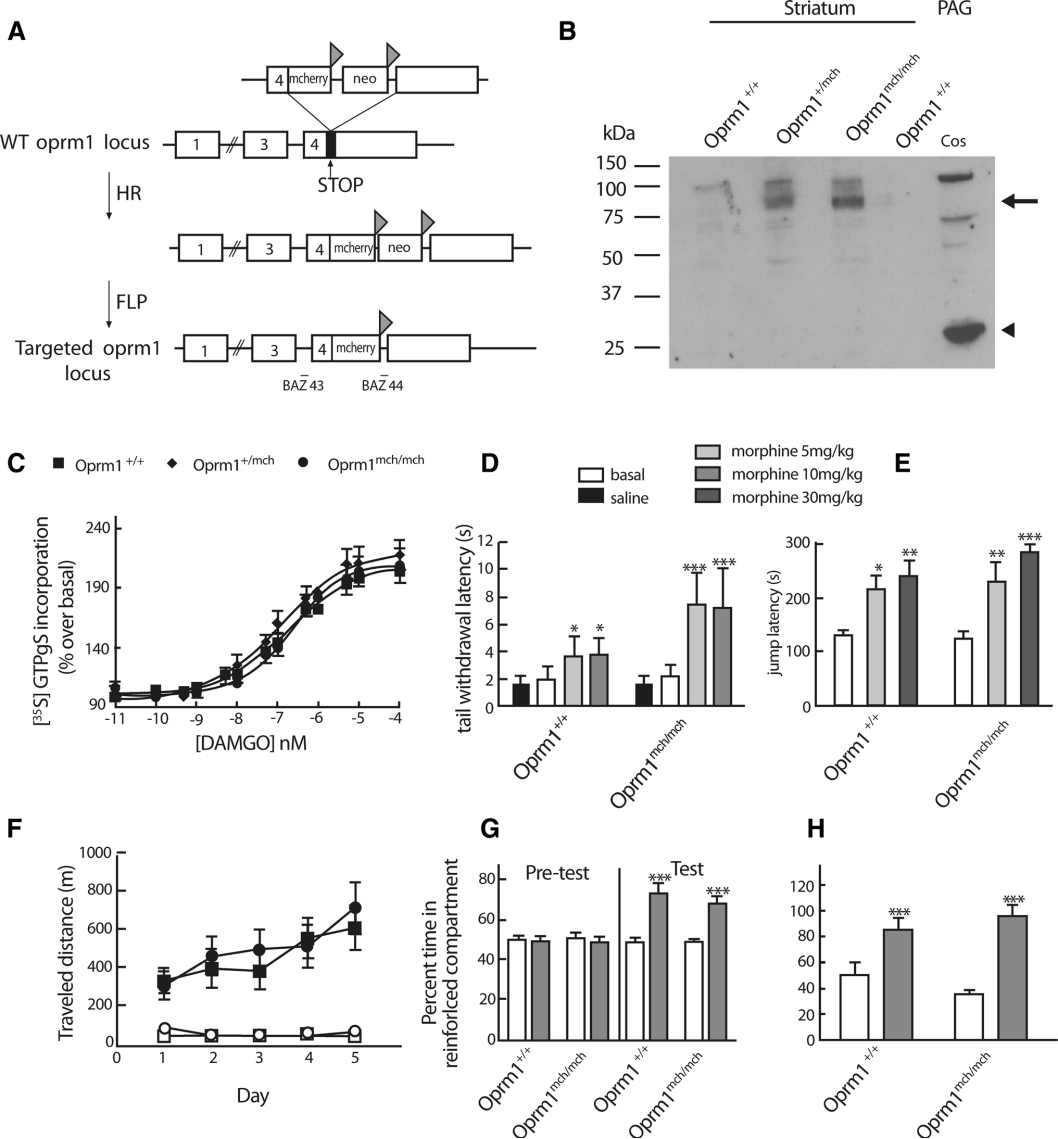
Expression of functional receptors in MOR-mcherry knock-in mice. **a** Targeting strategy: *Oprm1* exons, mcherry cDNA, and the FRT (*triangle*) flanked neomycin cassette are, respectively, displayed as *exon number*, *mcherry*, and *neo*. Homologous recombination (HR) was followed by FLP recombinase treatment (FLP) in ES cells. Positions of the oligonucleotides (BAZ 43, BAZ 44) used for genotyping are indicated. **b** Western blot: detection of MOR-mcherry fusion by immunoblotting with antibodies directed against mcherry on membranes from striatum and periaqueductal gray (PAG) from wild-type (*Oprm1* ^+/+^), heterozygote (*Oprm1* ^+/mch^) and homozygote (*Oprm1* ^mch/mch^) mice (MOR-mcherry fusion indicated by *arrow*). Cos cells transfected with a plasmid encoding mcherry (cos) were added as a control for unbound mcherry protein detection with the anti-mcherry antibody (*arrow head*). **c** G protein activation: similar [^35^S] GTPγS incorporation was measured on brain membranes from wild-type (*filled square*) (*n* = 8), heterozygous (*n* = 7) (*filled diamond*) and homozygous (*n* = 11) (*filled circle*) mice following stimulation with the mu-selective agonist DAMGO. Data are the mean ± sem from independent experiments performed in triplicate (*n* = 3 animals per genotype). **d** Tail immersion test: similar tail withdrawal latencies were measured at 52 °C in wild-type (*Oprm1* ^+/+^) and MOR-mcherry (*Oprm1* ^mch/mch^) mice after saline or morphine injection (5 or 10 mg/kg, i.p.). Data are presented as mean ± sem (*n* = 16 animals/group). *p < 0.05, ***p < 0.001 morphine effect compared to baseline. **e** Hot plate test: similar jump latencies from a hot plate at 52 °C were measured in wild-type (*Oprm1* ^+/+^) and MOR-mcherry (*Oprm1* ^mch/mch^) mice after saline or morphine injection (5 or 10 mg/kg, i.p.). Data are presented as mean ± sem (*n* = 16 animals/group). *p < 0.05, **p < 0.01, ***p < 0.001 morphine effect compared to baseline. **f** Locomotor sensitization: wild-type (*Oprm1* ^+/+^) or MOR-mcherry (*Oprm1* ^mch/mch^) mice received daily morphine (25 mg/kg, i.p.) or saline injections for 5 days. Similar locomotor activities were recorded for 1 h. Data are expressed as total traveled distance (mean ± sem) (*n* = 8–10 animals/group). **g** Conditioned place preference: wild-type (*Oprm1* ^+/+^) or MOR-mcherry (*Oprm1* ^mch/mch^) mice showed similar preference for the compartment associated with morphine (10 mg/kg, s.c.) following three conditioning sessions. Place preference corresponds to the time spent in the drug-paired compartment expressed as a percentage of time spent in the two compartments during the 20 min pre- and post-test conditioning sessions (*n* = 8 animals/group). Data are presented as mean ± sem. Treatment effect ***p < 0.001. **h** Physical dependence: global scores of pharmacological withdrawal precipitated by naloxone (1 mg/kg, s.c.) were similar in wild-type (*Oprm1* ^+/+^) or MOR-mcherry (*Oprm1* ^mch/mch^) mice treated with escalating doses of morphine (20, 40, 60, 80, 100 mg/kg) or in saline-treated controls (*n* = 8/group). Data are presented as mean ± sem. Drug effect ***p < 0.001

DOR-eGFP knock-in mice expressing the delta opioid receptor fused to its C terminus to a green fluorescent protein were generated by homologous recombination. In these mice, the eGFP cDNA was introduced into exon 3 of the delta opioid receptor gene, in frame and 5′ from the stop codon (Scherrer et al. 2006). MOR-mcherry knock-in mice expressing the mu opioid receptor fused its C-terminus to the red protein mcherry were generated by homologous recombination following a procedure similar to the one used for DOR-eGFP knock-in mice. A targeting construct in which the *Oprm1* stop codon has been replaced by a Gly-Ser-Ile-Ala-Thr-mcherry encoding cDNA followed by a neomycin resistance gene flanked by FRT sites was transfected into ES cells (Fig. 1). Two independent homologous recombinants were electroporated with a FLP recombinase expressing plasmid to excise the neomycin gene and microinjected into C57Bl6/J blastocysts. Chimeric mice were crossed with C57Bl6/J mice to obtain F1 heterozygous progenies. Heterozygous animals were intercrossed to generate mice homozygous for *Oprm1*-mcherry that are fertile and develop normally. DOR-eGFP mice were crossed with MOR-mcherry mice to obtain mice homozygous for both constructs. Wild-type mice were used as control in behavioral experiments. The genetic background of all mice was C57/Bl6/J: 129svPas (50:50 %). Mice genotyping was performed by standard PCR technique using a 5′ oligonucleotide located on the fourth exon of the *oprm1* gene (BAZ 43 tgacgtgacatgcagttgagattt) and a 3′ oligonucleotide located in the 3′ UTR untranslated region (BAZ 44 tcccacaaaccctgacagcaac). Introduction of the coding sequence for mcherry increased the size of the amplified fragment by about 800 bp enabling identification of wild type *oprm1* ^+*/*+^, heterozygous *oprm1* ^+/mch^ and homozygous *oprm1* ^mch/mch^ animals by PCR.

Mice were housed in a temperature- and humidity-controlled animal facility (21 ± 2 °C, 45 ± 5 % humidity) on a 12-h dark–light cycle with food and water ad libitum. Male and female mice aged 8–14 weeks were used in all protocols. All the experiments were conducted during the light period. All experiments were performed in accordance with the European Communities Council Directive of 26 May 2010 and approved by the local ethical committee (Com’Eth 2012-006).

### Drugs

Morphine chlorhydrate (Francopia, Lyon, France) was administered s.c. or i.p. at doses of 5, 10, 25 or 30 mg/kg. Naloxone hydrochloride (Sigma) was used at 1 mg/kg (s.c.) for the pharmacological induction of morphine withdrawal. SNC 80 (Tocris) was used in vivo at 10 mg/kg (s.c.). All drugs were administered at 10 mL/kg and dissolved in 0.9 % NaCl (solution used for control animals).

The delta agonist AR-M100390 (*N*, *N*-diethyl-4-(phenyl-piperidin-4-ylidenemethyl)-benzamide) is a SNC80 derivative synthesized at AstraZeneca R&D Montreal (Canada). The kappa agonist U50-488H (2-(3,4-dichlorophenyl)-*N*-methyl-*N*-[(2R)-2-pyrrolidin-1-ylcyclohexyl] acetamide) was purchased from Sigma. [^3^H] Diprenorphine (50 Ci/mmol), [^3^H] DAMGO (35 Ci/mmol) and [^35^S] GTPγS (1,250 Ci/mmol) were from Perkin Elmer Life and Analytical Sciences (Boston, MA, USA).

### Antibody characterization

Rabbit polyclonal antibodies raised against eGFP (Cat. Nr A-6455, Molecular Probes, Paisley, UK, dilution 1:1,000), mcherry (Cat Nr 632496, Clontech, dilution 1:1,000) were used for fluorescent protein detection when indicated. MORs were detected using a rabbit polyclonal antibody raised against the C-terminus (1:100, generous gift from Dr C. Evans). Primary antibodies used for co-localization with neuronal markers are mouse monoclonal antibodies raised against calbindin D-28K (Cat. Nr 300, Swant, Bellinzona, Switzerland, dilution 1:1,000), or parvalbumin (Cat. Nr 235, Swant, Bellinzona, Switzerland, dilution 1:1,000), rat monoclonal antibodies raised against somatostatin (Cat. Nr MAB 354, Millipore, Billerica, MA, USA, dilution 1:1,000). The following AlexaFluor-conjugated secondary antibodies (Molecular Probes, Paisley, UK) were used: goat anti rabbit AlexaFluor 488 conjugated (Cat. Nr A-11034, dilution 1:2,000), goat anti rabbit IgG AlexaFluor 594 conjugated (Cat. Nr A-11012, dilution 1:2,000), goat anti mouse IgG AlexaFluor 594 conjugated (Cat. Nr A-11005, dilution 1:500), goat anti rat IgG AlexaFluor 594 conjugated (Cat. Nr 1-11007, dilution 1:500), goat anti mouse IgG AlexaFluor 350 conjugated (Cat. Nr 1-21049, dilution 1:500). Absence of cross-reactivity (rabbit/mouse, rabbit/rat, mouse/rat) was systematically checked in control experiments for each antibody. Immunohistochemistry was also performed without primary antibodies to verify absence of non-specific staining by the secondary antibody alone.

### Real-time quantitative PCR analysis

RNA extraction, cDNA synthesis and qPCR assays were carried out as described previously (Befort et al. 2008). Total brain tissue was collected from three animals for each genotype to isolate RNA using Trizol reagent (Invitrogen, Cergy Pontoise, France) according to the manufacturer’s instructions. Briefly, 2.5 μg of total RNA was reverse transcribed using Superscript II (200 or 400 U, Invitrogen, Cergy Pontoise, France) with anchored-oligodT primer (8 mmol/L), random Hexamer (16 mmol/L), and deoxynucleotide triphosphates (500 μmol/L each). Real-time PCR was performed in triplicate on a MyIQ BioRad instrument using iQSYBRGreen supermix (Bio-Rad, Marnes-la-Coquette, France), cDNA (0.5 μL), and gene-specific primers (200 nmol/L) in a 25 μL reaction as recommended by the manufacturer. Gene-specific primers were designed using Primer3 (http://frodo.wi.mit.edu/primer3/). Sequences of primers are provided below. Thermal cycling parameters were 2 min at 95 °C followed by 40 cycles of 15 s at 95 °C, 15 s at 60 °C and 30 s at 72 °C. Relative expression ratios were normalized to the level of HPRT reference gene, and the 2-DDCt method was used to evaluate the differential expression level. Two reference genes (β-actin, Rplp0) were tested in each run as an internal control.

**Table:** Primers for RT PCR

Gene	Forward	Reverse	Target sequence
mMOR	GAGCCACAGCCTGTGCCCT	CGTGCTAGTGGCTAAGGCATC	Exon1/exon2
mDOR	GCTCGTCATGTTTGGCATC	AAGTACTTGGCGCTCTGGAA	Exon1/exon2
mKOR	CCTGGCATCATCTGTTGGTA	GGAAACTGCAAGGAGCATTC	Exon2/exon3
HPRT	TGACACTGG**T**AAAACAATGCA	GGTCCTTTTCACCAGCAAGCT	
Rplp0	TGAGATTCGG GATATGCTGTTG	TTCAATGGTGCCTCTGGAGAT	
β-Actin	GACGGCCAGGTCATCACTAT	CCACCGATCCACACAGAGTA	

### Ex vivo tissue analysis of MOR-mcherry mice

Membrane preparations were carried out as described previously (Befort et al. 2001). Briefly, whole brain were removed, immediately frozen on dry ice, and stored at −80 °C prior to use. Whole brain membranes were prepared by homogenizing the brain in ice-cold 0.25 M sucrose solution 10 vol (ml/g wet weight of tissue). Samples were then centrifuged at 1,100*g* for 10 min. Supernatants were collected and diluted five times in buffer containing 50 mM Tris–HCl (pH 7.4) and 1 mM EDTA, following which they were centrifuged at 35,000*g* for 30 min. The pellets were homogenized in 2 ml ice-cold sucrose solution (0.32 M) and aliquots kept at −80 °C until further use.

#### Scatchard analysis

50 μg of membrane proteins was incubated in the presence of variable concentrations (3 10^−9^ to 2 10^−10^ M) of [^3^H] DAMGO for 1 h at 25 °C. Membranes were washed and filtered, and radioactivity was quantified using a liquid scintillation counter. Assays were performed in triplicates in eight experiments using six different membrane preparations.

#### [^35^S] GTPγS binding assay

5 μg of membrane proteins was used per well. Samples were incubated with the mu agonist DAMGO, the delta agonist AR-M1000390 or the kappa agonist U50-488H (10^−4^ to 10^−11^ M) for 1 h at 25 °C in assay buffer 50 mM Tris–HCl (pH 7.4), 3 mM MgCl_2_, 100 mM NaCl, 0.2 mM EGTA containing 30 μM GDP and 0.1 nM [^35^S] GTPγS. Incubation was terminated by rapid filtration and washing in ice-cold buffer (50 mM Tris–HCl, 5 mM MgCl_2_, 50 mM NaCl, pH 7.4). Bound radioactivity was quantified using a liquid scintillation counter. Non-specific binding was defined as binding in the presence of 10 μM GTPγS, and basal binding was assessed in the absence of agonist. Assays were performed in triplicates in nine experiments using six different membrane preparations.

#### Co-immunoprecipitation

Membrane preparations (500 μg) were solubilized in Tris–HCl 50 mM pH 7.4, 100 mM NaCl, 10 % CHAPS, complete protease inhibitor cocktail (Roche applied Bioscience, Mannheim, Germany) for 1 h at 4 °C, immunoprecipitated with either 1 μg anti-eGFP or 1 μg anti-mcherry antibodies for 1 h at 4 °C and isolated by incubation with G protein Sepharose for 1 h at 4 °C. Samples were washed three times with Tris–HCl 50 mM pH 7.4 and resuspended in SDS-PAGE sample buffer.

#### Western blot analysis

Total protein content of brain membranes was determined by Bradford assay. Samples were heated in loading buffer (62.5 mM Tris–HCl, pH 6.8, 5 % (wt/vol) ß-mercaptoethanol, 2 % (wt/vol) SDS, 10 % (vol/vol) glycerol, 0.1 % (wt/vol) Bromophenol blue) for 5 min at 95 °C. 50 µg proteins were loaded onto an 8 % SDS-PAGE gel. Proteins were transferred onto Immobilon P polyvinylidene difluoride (PVDF) membrane (Millipore, Billerica, MA, USA). Following blocking in 5 % (wt/vol) non-fat dry milk in 50 mM Tris–HCl pH 8, 150 mM NaCl, 0.2 % (vol/vol) Tween 20 (TBST) for 1 h, PVDF membranes were incubated overnight at 4 °C with a 1:1,000 dilution of the anti mu opioid receptor or a 1:1,000 dilution of the anti mcherry antibody. PVDF membranes were washed three times for 10 min with 5 % (wt/vol) non-fat dry milk in TBST, incubated for 2 h with a 1: 10 000 dilution of HRP-conjugated anti-mouse (Fab′_2_) fragment antibody in 5 % (wt/vol) non-fat dry milk in TBST. PVDF membranes were washed three times for 10 min in TBST. Chemiluminescence was detected using ECL^+^ according to the manufacturer’s instructions.

### Behavioral testing

Experiments were performed in stable conditions: 21 ± 2 °C, 45 ± 5 % humidity, 40 ± 2 lux. All experiments were preceded by 2 days of animal handling. Tail immersion and hot plate tests were used to evaluate antinociceptive responses.

#### Tail immersion test

The mouse was maintained in a cylinder and the tail immersed into a heated water bath set at 52 °C. Morphine (5 or 10 mg/kg) or a saline solution were injected i.p. Tail withdrawal latencies were measured 45 min later with a 10 s cutoff time. Baseline responses were measured 1 h prior drug injection.

#### Hot plate test

Morphine (5 or 10 mg/kg) or a saline solution was injected i.p. The mouse was placed on a 52 °C hot plate 45 min later and latencies to jump were recorded with a 300 s cutoff time.

#### Conditioned place preference test

##### Apparatus

Place conditioning experiments were performed in unbiased computerized boxes (Imetronic, Pessac, France) formed by two Plexiglas chambers (15.5 × 16.5 × 20 cm) separated by a central alley (6 × 16.5 × 20 cm). Two sliding doors (3 × 20 cm) connected the alley with the chambers. Two triangular prisms of transparent polycarbonate were arranged in one chamber, and one rectangular prism in the other to form different shape patterns (covering the same surface). Distinct-textured removable floors made of translucent polycarbonate provided additional contextual cues. The activity and location of mice were recorded using five photocells located throughout the apparatus. Behavioral data were collected by an interface connected to a PC. Light intensity in the chambers was set at 30 Lux.

##### Experimental protocol

Animals were naive when conditioning started. Morphine conditioning consisted of 3 phases. On day 1, naive mice were placed in the central alley and allowed to freely explore the apparatus for 20 min for a pretest session. Based on the individuals’ spontaneous preference during this pretest phase, the drug-paired chamber was assigned in such a way that saline and morphine groups were counterbalanced and unbiased toward contextual cues. Statistical analysis on pre-test data indicated no bias between the two chambers (*p* = 0.99). Conditioning phase lasted 3 days. Mice underwent two daily conditioning sessions, vehicle and drug paired, 7 h apart. Drug pairings were performed in the morning (10:00 AM). The animals were injected with either morphine (10 mg/kg, s.c.) or saline (controls) immediately before being confined in the “drug-paired” chamber. Vehicle pairings were performed in the afternoon (4:00 PM). All the animals received an injection of saline and were confined in the vehicle-paired compartment. Testing phase was conducted on day 5. The animals, in a drug-free state, were placed in the neutral central alley and allowed to explore the apparatus for 20 min with the two sliding doors opened. The time spent in each chamber was recorded.

#### Naloxone-precipitated withdrawal

Mice daily received escalating doses (20, 40, 60, 80, 100 mg/kg) morphine i.p. or a saline solution for 6 days. Physical dependence to morphine was verified by measuring withdrawal syndrome precipitated by a naloxone (1 mg/kg, s.c.) injection 2 h after the last morphine injection. A global withdrawal score was calculated as previously described (Berrendero et al. 2003).

#### Locomotor sensitization

Locomotor activity was assessed in clear Plexiglas boxes (21 × 11 × 17 cm) placed over a white Plexiglas infrared-lit platform. Light intensity of the room was set at 15 lux. The trajectories of the mice were analyzed and recorded via an automated tracking system equipped with an infrared-sensitive camera (Videotrack; View Point, Lyon, France). Behavioral testing started when the animals were placed in the activity boxes for a 60-min habituation period. They were then injected with saline and locomotor activity was measured for another 1  h. Animals were then injected with morphine (25 mg/kg) or saline and activity was measured for 2  h. Locomotor activity was assessed during five consecutive days (Contet et al. 2008).

### Tissue preparation and immunohistochemistry

Mice were anesthetized with ketamine/xylazine (100/10 mg/kg, i. p.) and perfused intracardiacally with 50 ml of 4 % paraformaldehyde (PFA) (at 2–4 °C) in PB 0.1 M or PBS 1X (Dulbecco’s Phosphate Buffer Saline, Sigma Aldrich), pH 7.4. Brains were post-fixed for 24 h at 4 °C in 4 % PFA solution, cryoprotected at 4 °C in a 30 % sucrose, PB 0.1 M pH 7.4 solution, embedded in OCT (Optimal Cutting Temperature medium, Thermo Scientific), frozen and kept at −80 °C. 30-μm thick brain sections were cut with a cryostat (CM3050, Leica) and kept floating in PB 0.1 M pH 7.4.

Immunohistochemistry was performed according to standard protocols (Erbs et al. 2012). Briefly, 30-μm thick sections were incubated in blocking solution (PB 0.1 M pH 7.4, 0.5 % Triton X100 (Sigma, St. Louis, MO, USA), 5 % normal goat or donkey serum (Invitrogen, Paisley, UK) depending on the secondary antibody) for 1 h at room temperature (RT). Sections were incubated overnight at 4 °C in the blocking solution with appropriate primary antibodies. Sections were washed three times with PB 0.1 M pH 7.4, 0.5 % Triton X100, incubated for 2 h at RT with appropriate AlexaFluor-conjugated secondary antibodies. Sections were washed three times and mounted on SuperfrostTM glass (Menzel-Glaser) with Mowiol (Calbiochem, Darmstadt, Germany) and 4′, 6-diamidino-2-phenylindole (DAPI) (Roche Diagnostic, Mannheim, Germany) (0.5 μg/ml).

DOR-eGFP fluorescence was enhanced by detection with an anti-GFP antibody and a secondary antibody coupled to the AlexaFluor 488. MOR-cherry fluorescence was enhanced by detection with an anti-mcherry antibody and a secondary antibody coupled to AlexaFluor 594. Double labeling was performed to co-localize DOR-eGFP or MOR-mcherry with the chosen neuronal marker. Antibodies specific for the neuronal markers were detected with a secondary antibody coupled to the AlexaFluor 594 or 488 depending on amplification of the DOR-eGFP or MOR-mcherry signal, respectively, or with secondary antibody coupled to the AlexaFluor 350 for triple labeling.

### Immunocytochemistry on MOR-mcherry primary neuronal cultures

Primary neuronal cultures were performed as previously described (Pradhan et al. 2009). Briefly, P0 mice pups were decapitated, and hippocampi were dissected and digested with papain (15 U/ml, Worthington). Cells were plated on glass coverslips coated with poly-l-lysine (PLL, Sigma) in B27/NeurobasalA medium (Invitrogen) completed with 0. 5 mM glutamine and antibiotics. Cells were plated at a density of 8 × 10^4^ cells/cm^2^. Medium was replaced 60 min after plating, and half the medium changed every 5–7 days. Cultures were maintained for 15 days in vitro (DIV). Fully matured primary neurons (DIV 10–14) were used for DAMGO-induced receptor internalization studies. Cells were fixed with 4 % PFA in PBS before or at various time points after 1 μM DAMGO addition. Immunological detection with an anti-mcherry antibody was then performed as described previously (Massotte 2006). Briefly, cells were incubated in blocking solution (PB 0.1 M pH 7.4, 0.2 % Tween 20 (Sigma, St. Louis, MO, USA), 5 % normal goat serum (Invitrogen, Paisley, UK) for 1 h at room temperature (RT). Coverslips were incubated overnight at 4 °C in the blocking solution with anti-mcherry antibodies (1:1,000), washed three times with PB 0.1 M pH 7.4, 0.2 % Tween 20 and incubated for 2 h at RT with goat anti rabbit AlexaFluor 594-conjugated secondary antibodies. Coverslips were washed three times and mounted with Mowiol (Calbiochem, Darmstadt, Germany) and 4′, 6-diamidino-2-phenylindole (DAPI) (Roche Diagnostic, Mannheim, Germany) (0.5 μg/ml).

### Image acquisition

Image acquisition was performed with the slide scanner NanoZoomer 2 HT and fluorescence module L11600-21 (Hamamatsu Photonics, Japan). The light source LX2000 (Hamamatsu Photonics, Japan) consisted in an ultra high-pressure mercury lamp coupled to an optical fiber. Single RGB acquisition was made in the epifluorescence mode with the 3-chip TDI camera equipped with a filter set optimized for DAPI, fluorescein and tetramethylrhodamine detection. The scanner was equipped with a time delay integration camera and performed line scanning that offered fast acquisition at high resolution of the fluorescent signal. The acquisition was performed using a dry 20× objective (NA 0.75). The 40× resolution was achieved with a lens converter. The latter mode used the full capacity of the camera (resolution 0.23 μm/pixel). Neurons expressing a given fluorescent marker are visualized using the NDP viewer system with an integrated high-resolution zoom and possibility to separate the different fluorescent components.

Observations with a confocal microscope (SP2RS, Leica) using 40× (NA 1.25) and 63× (NA 1.4) oil objectives were used to validate mu and delta opioid receptor co-localization. Images were acquired with the LCS (Leica) software. Confocal acquisitions were performed in the sequential mode (single excitation beams 405, 488 and 568 nm) to avoid potential cross talk between the different fluorescence emissions.

Brain regions were identified using the Mouse Brain Atlas (2nd edition) from G. Paxinos and K.B.J. Franklin.

Images corresponding to each brain section were individualized using the NDP toolkit program (Hamamatsu Photonics, Japan) and arranged according to the rostrocaudal axis for coronal sections and lateromedial axis for the sagittal sections.

### Statistical analysis

Statistical analysis was performed with Graph-Pad Prism v4 (GraphPad, San Diego, CA) and Statistica v9 (StatSoft, Maisons-Alfort, France). In vitro pharmacology experiments were analyzed using a one-way ANOVA. Behavioral experiments were analyzed using a two-way ANOVA. Multiple comparisons were made using Newman-Keuls or Tukey tests for post hoc analysis. A paired *t* test was performed to verify that the apparatus used in the conditioned place preference test was unbiased. Place conditioning data were expressed as percentage of time spent in the drug-paired compartment. Four-way ANOVA was performed with gender, genotype and treatment as between-group factors and conditioning (pretest versus test session) as a within-group factor.

## Results

### Generation and characterization of MOR-mcherry knock-in mice

We generated MOR-mcherry knock-in mice expressing MOR in fusion with the red fluorescent protein mcherry at the C terminus (Fig. 1a), as previously done for DOR and the green fluorescent protein eGFP (Scherrer et al. 2006). DNA sequencing showed accurate insertion of the mcherry cDNA at genomic level in homozygous mutant mice (*Oprm1* ^mch/mch^ or MOR-mcherry mice). Quantitative mRNA analysis revealed that the genomic modification does not disrupt *Oprm1* transcription, which was slightly increased in knock-in animals similarly to DOR-eGFP knock-in mice (Scherrer et al. 2006) (online resource Fig. 1). Western blot analysis of brain tissue using antibodies recognizing mcherry showed expression of a protein with expected molecular mass for the fusion construct, and no free mcherry protein could be detected (Fig. 1b). Scatchard analysis of [^3^H] DAMGO binding to brain membranes from *Oprm1* ^+/+^, *Oprm1* ^+/mch^ and *Oprm1* ^mch/mch^ animals showed similar ligand affinity (Kd 0.65 ± 0.12, 0.51 ± 0.16 and 0.51 ± 0.07 nM, respectively) and receptor density (207 ± 39, 230 ± 41 and 293 ± 18 fmol/mg, respectively, *p* = 0.223). Further, the MOR-selective agonist DAMGO activated G proteins in brain membranes from *Oprm1* ^+/+^, *Oprm1* ^+/mch^ and *Oprm1* ^mch/mch^ mice with similar potency (184 ± 33, 120 ± 27 and 184 ± 27 nM, respectively) and maximal efficacy (210 ± 11, 196 ± 16 and 199 ± 12 %, respectively) (Fig. 1c). Binding and signaling properties of DOR (AR-M1000390) and KOR (U50-488H) agonists were otherwise unchanged in mutant mice (online resource Fig. 1).

Next, we compared well-described behavioral effects of morphine in *Oprm1* ^mch/mch^ mice and their wild-type *Oprm1* ^+/+^ controls. Thermal antinociception following acute morphine administration was identical in animals from the two genotypes using both tail immersion and hot plate tests at two doses (5 and 10 mg/kg, s.c.) (Fig. 1d). A single injection of morphine (25 mg/kg, i.p.) also produced comparable locomotor activation in the two genotypes, and sensitization to this effect developed likewise upon repeated injections for 5 days (Fig. 1e). Reinforcing effects of morphine (10 mg/kg, s.c.) were tested in a conditioned place preference paradigm. *Oprm1* ^mch/mch^ and *Oprm1* ^+/+^ mice displayed similar marked preference for the morphine-paired chamber after conditioning (gender effect: *F*_1,24_ = 2.08, NS; genotype effect: *F*_1,24_ < 1; treatment effect: *F*_3,24_ = 46.26, *p* < 0.0001; conditioning effect: *F*_1,24_ = 43.24, *p* < 0.0001) (Fig. 1f). Finally, mice were injected daily with morphine (30 mg/kg s.c. for 6 days) and comparable physical withdrawal was measured in the two genotypes upon naloxone injection (1 mg/kg i.p.) (Fig. 1g). Altogether, data demonstrate that functional properties of MOR are maintained in MOR-mcherry mice both in vitro and in vivo, as previously observed for DOR-eGFP knock-in mice (Scherrer et al. 2006; Pradhan et al. 2009).

### Receptor subcellular localization in MOR-mcherry knock-in mice

**Fig. 2:**
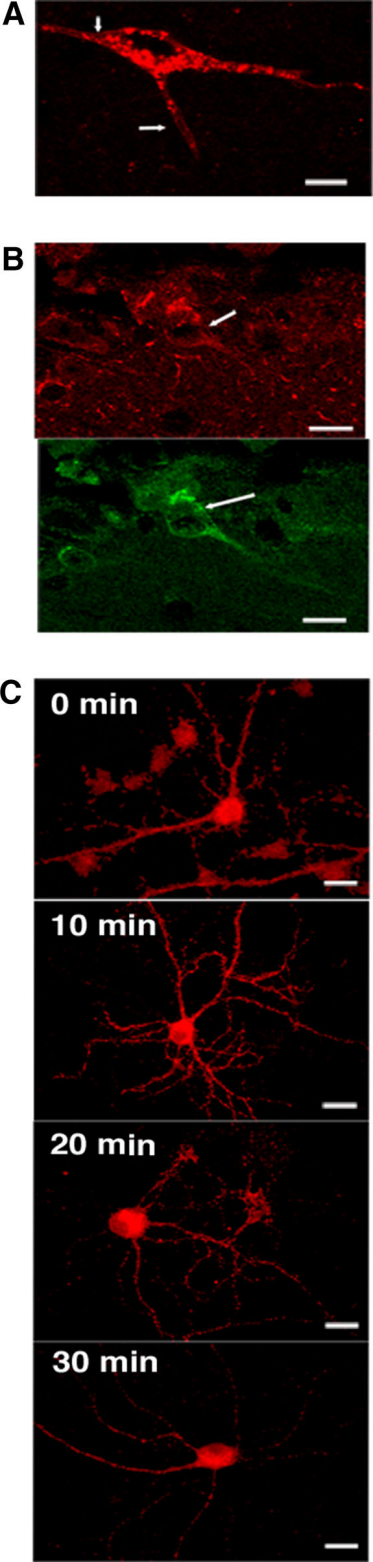
MOR-mcherry subcellular localization and trafficking. **a** The in vivo fluorescent signal associated to MOR-mcherry is located at the surface of the neuron (*white arrow*) and intracellularly. **b** In vivo localization of MOR-mcherry at the plasma membrane (*white arrow*) upon detection with an anti-mcherry receptor antibody revealed with an AlexaFluor 594-coupled secondary antibody (*top*) or upon detection with an anti-mu receptor antibody revealed with an AlexaFluor 488-coupled secondary antibody (*bottom*). **c** MOR-mcherry subcellular localization in primary hippocampal neurons fixed at various time points after stimulation with the MOR-selective agonist DAMGO 1 μM. *Scale bars* 10 μm

Unlike DOR-eGFP that predominantly localizes at the plasma membrane under basal conditions (Scherrer et al. 2006; Pradhan et al. 2009), the MOR-mcherry fluorescent signal was strong inside neurons (Fig. 2a) and rather weak at the plasma membrane. Because fusion to mcherry may alter receptor distribution, notably the inside/outside receptor ratio, we compared the cellular distribution of MOR-mcherry to that of the native receptor using heterozygous *Oprm1* ^+/mch^ animals co-expressing the two receptor forms. Double labeling using antibodies raised against mcherry or MOR demonstrated overlapping patterns with a similar weak signal at the neuronal surface (Fig. 2b). This result indicates that the low amount of cell surface MOR-mcherry does not result from deficient receptor trafficking, but rather reflects the genuine distribution of the native receptor. This is consistent with the previous reports describing substantial intracellular localization of endogenous untagged receptors (Poole et al. 2011). In addition, the intensity of the intracellular fluorescent signal varied across brain regions. Combined with the observation of intact in vivo morphine responses in MOR-mcherry mice (Fig. 1), our data strongly suggest that, under physiological conditions, only a small proportion of MOR is present at the cell surface, and that this distribution is compatible with full morphine effects. Electron microscopy showed that intracellular fluorescence is present in large multivesicular bodies suggesting that intracellular proteins are at least in part involved in the degradative pathway (not shown).

We finally examined whether MOR-mcherry internalizes upon agonist treatment, as largely described for MOR expressed in transfected cells (Borgland et al. 2003) or neurons (Arttamangkul et al. 2008; Haberstock-Debic et al. 2005). Although strong intracellular expression of MOR-mcherry hampers easy detection of receptor trafficking, internalization was detectable in primary hippocampal neurons from MOR-mcherry mice upon DAMGO exposure (Fig. 2c). A typical internalization punctate pattern was visible after 10 min, a time when surface staining had entirely disappeared, and the distribution returned to the basal pattern after 30 min. MOR-mcherry therefore shows normal trafficking response with kinetics similar to previous reports for the untagged receptor expressed in neurons (Rodriguez-Munoz et al. 2007; Trafton et al. 2000).

### Neuroanatomy of MOR-mcherry and DOR-eGFP in double mutant mice

**Fig. 3:**
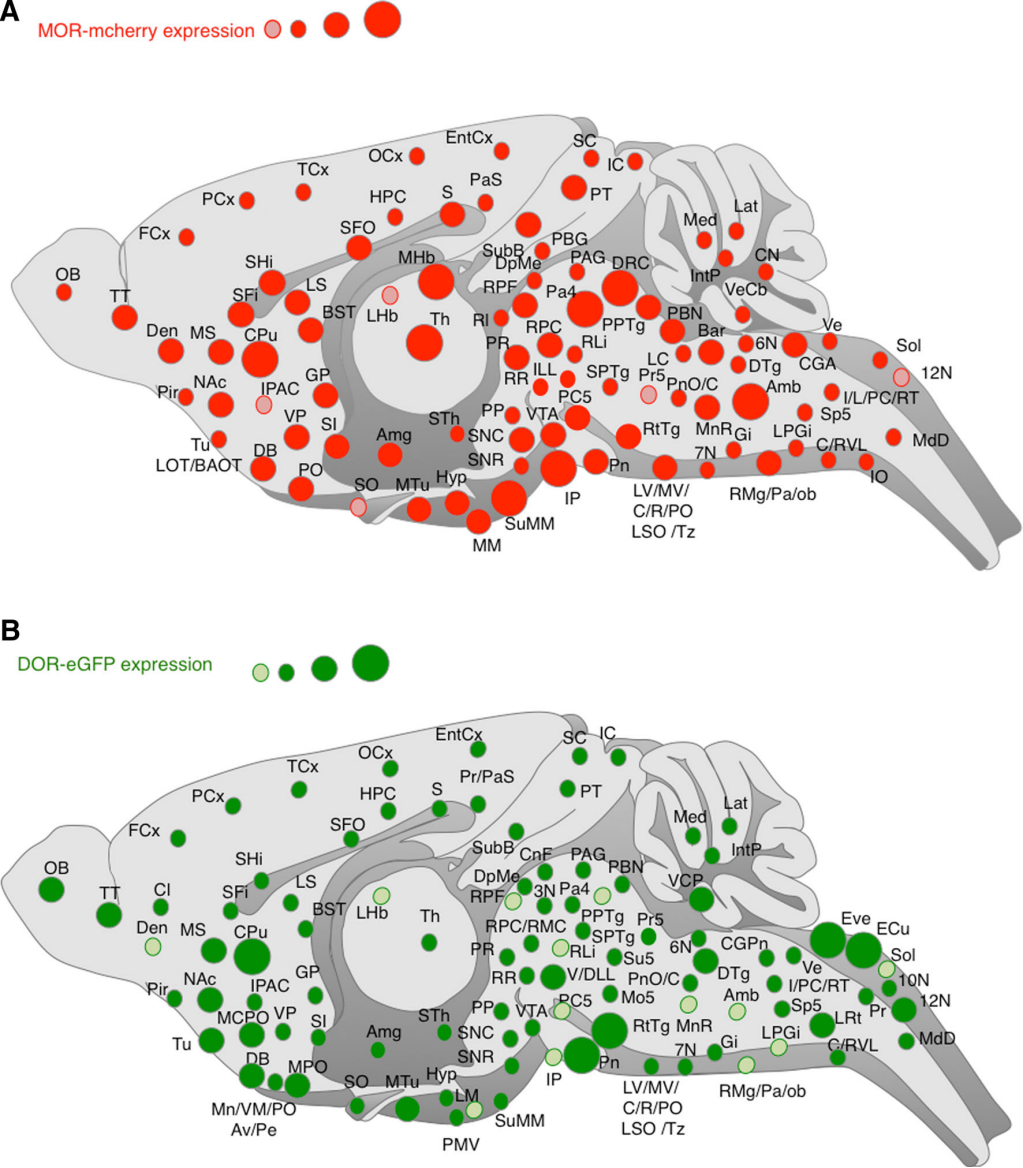
Distribution of mu and delta opioid receptors in the nervous system. **a** Brain distribution of the MOR-mcherry construct. The size of the *red circle* is indicative of the abundance of the receptor in the given area. A *pink circle* indicates low expression level. **b** Brain distribution of the DOR-eGFP construct. The size of the *green circle* is indicative of the abundance of the receptor in the given area. A *pale green circle* indicates low expression level. See list for abbreviations

MOR-mcherry and DOR-eGFP mice were crossed and the two fluorescent signals mapped throughout the brain, spinal cord and DRGs. Data are presented as an interactive virtual atlas accessible at http://mordor.ics-mci.fr/ and summarized in Fig. 3 and Table 1. MOR-mcherry was more readily visualized in cell bodies than neural processes, because of the high intracellular/extracellular protein ratio. In contrast, DOR-eGFP was predominantly seen at the plasma membrane, therefore receptor expression in neurites or passing fibers was better detected for DOR-eGFP than for MOR-mcherry.

**Table 1:** Relative distribution of MOR-mcherry and DOR-eGFP signals in the brain

	Mu	Delta		Mu	Delta			Mu	Delta
**Olfactory structures**			**Dorsal thalamus**				**Brainstem**		
Accessory olfactory bulb			Anterior group				Interpeduncular nucleus		
EPlA	++	+	AD	+	+/−		IPC	++	+/−
GrA	−	+	IAD	++	+		IPI	+	+/−
MiA	++	+	AVDM	+/++	++		IPL	+++	+/−
Olfactory bulb			AVVL	−	+		IPR/IPDM/IPRL	+++	+/−
Epl	−	+++	AM	+	++		Oculomotor		
Gl	+	−	IAM	+	++		3N	−	+
GrO	−	++	Lateral group				EW	−	+
IPl	−	+	LDVL	−	+/−		Dk	−	+
Mi	++	++	LDDM	−	+		InC	−	+
Anterior olfactory nucleus			LDMM	−	+		MA3	−	+
AOE	+++	+	Ventral group				RI	+	+
DTT	++	++	VA	+	+		Su3/Su3C	+	+/−
VTT	++	−	VL	−	+		4N	−	+
LOT	+	−	VM	++	+/−		Pa4	+++	+
Tu	−	++	VPM/VPL	−	+		Pr	−	+
**Cerebral cortex**			VPPC(Gus)	++	+		Periaqueductal gray		
Orbital	+/++	+/−	Medial group				DMPAG	−	+
FrA	+	++	MD	+	+/−		DLPAG	+	+/−
DP	−	++	MDC	+/−	+/−		LPAG	+/++	+
PrL	+	++	MDL	++	+/−		VLPAG	+	+
IL	+	++	MDM	+	+/−		Parabrachial nucleus		
Cg	+	++	Sub	−	+/−		LPBS	+	+/−
M1/M2	+	+	Lateral geniculate				LPBD	+/−	+/−
AI	++	++	DLG	−	−		LPBC	+/−	+
DI/GI	+	++	VLGMC	+	−		LPBV	+	+
Pir	+	+	VLGPC	−	−		LPBI	+	+
S1/S2	+/++	++	IGL	+/−	−		LPBE	++++	−
Ect	+	++	Medial geniculate				MPB	+	+
LEnt	+	+	MGD	−	−		MPBE	+	+
MEnt	+	+	MGV	−	−		Raphe		
RSA	+	−	MGM	+	+		RLi	+	+/−
Te + aud	+	−	SG	+	+		MnR	+/++	+/−
V1 + V2	−	+	Posterior group				PMnR	++	+/−
**Basal forebrain**			PLi	+	+/−		DRC	+++	+/−
Cl	−	+	PoT	++	+/−		DRV/DRI/DRD	+/−	+/−
ICjM	−	−	Eth	−	+		RMg/RPa/Rob	++	+/−
MS(Ld)	++	++	Reth	+/−	+/−		Red nucleus		
VDB	++	++	Midline group				PR	++	+
HDB	++	++	PVA	+++	−		RMC	−	+
LSD	−	+	PV	++	−		RPC	++	+
LSI	++	+	PVP	+/−	−		RPF	++	+/−
LSV	+	−	PT	+++	+		RR	+	+
TS	−	+	IMD	++	+/−		Reticular formation		
Shi	++	+	Re	+	+/−		CnF	−	+
SFi	++	+	VRe	++	+/−		DpMe	+	+
B (Meynert)	+	+	Rh	+++	−		PnC	+	++
SI	++	+	Xi	+++	−		PnO	+/−	+
VP	++	+	Intralaminar group				Gi	+	+
DEn	++	+/−	rCM	+++	+/−		DPGi	+/−	−
SFO	++	+	cCM	++	++		LPGi	+	+/−
**Basal ganglia**			CL	+++	+		GiA	+	+
AcbC	++	++	PC	++	+		GiV	+	+
AcbS	++	++	OPC	−	++		PCRtA	+/−	+
CPu	+++	+++	PIL	+	+		PCRt	+/−	+
LGP	++	+	PF	−	+		IRt	++	+
MGP	+	+	SPFPC	+/−	+/−		LRt	+/−	++
**Amygdala**			**Hypothalamus**				RVL	+/−	+
AAV	+	+	Periventricular				CVL	+	−
ACo	++	++	MnPO	−	+/−		MdD	+	+
PLCo	+	+	VMPO/AVPe/Pe	−	+		Tectum		
PMCo	++	+	PaLM	+/−	+		APT	+/−	+
APir	+	++	PaV/PaAP	+/−	−		PPT/MPT/OPT	++	+/−
AHi	+	+	SCh	−	−		SuG	+/−	−
CxA	+	+	SO	+/−	+		Op	+/−	−
AStr	+/−	+	Arc	+	+*/*++		InG	+	++
BAOT	+++	−	ME	−	+		InWh	+	++
LA	+/−	+	Medial				DpG	+/−	+
BLA	+/−	+++	MPA	++	+/−		PBG	+	−
BLP	+/−	++	MPOM/MPOL	++	++		CIC	++	+
BMA	++	+	AHA/AHC/AHP	+	+		ECIC	+	++
BMP	+	+	DM	++	+		DCIC	+/−	−
I	++	−	VMH	++	+		BIC	+	+
**Extended amygdala**			MTu	++	++		SubB	++	+/−
Central extended amygdala			Tu	+	+		Tegmentum		
BSTLD	+/++	+/−	LM	−	+/−		VTA	++	+
BSTLP/I/V	+	+/−	MM	++/+++	−		SNC	+/++	+/++
CeC	+	−	MMn	−	−		SNL	+	+
CeL	+/−	−	ML	+/−	−		SNR	+	+
CeI	+++	+	PMV/PMD	++	+		ATg/VTg	−	+
CeM	+/++	+	SuML	++	−		MiTg	+	++
IPAC	+/−	+	SuMM	+++	+		PPTg	++	+/−
Medial extended amygdala			PH	+	+		LDTg	+	+
BSTMA	++	+	Lateral				SPTg	+	+
BSTMP	++	+	MCPO	+/−	+		RtTg	++	+++
BSTMV	++	+/−	LPO	+	+/−		DTgC	+	++
MeAD	+++	+	LH	++/+++	+		DTgP	−	−
MeAV	++	+	PeF	+	+		DMTg	+	+
MePD	++	+	PSTh	+	+		PDTg	−	++
MePV	+	+	**Cerebellum**				Trigeminal		
BSTIA	+	+	CbCx	−	−		Me5	−	+
**Hippocampal formation**			IntP	+	++		Mo5	−	+
DG	+	+	Lat	+	+		PC5	++	+/−
CA1/CA3	+	+	Med	+	+		Su5/I5	+/−	+
Py	+	+	mcp	+	++		Pr5VL	+/−	−
S	++	+	**Brainstem**				Pr5DM	+/−	+
alv	+	+	Auditory system				Sp5	+	+
df	++	−	CPO	+/−	+		Vestibular		
fi	−	++	DPO	+/−	++		MVePC	+	+
PaS	+	+	RPO	++	+		MVeMC	+	+
PrS	−	++	MVPO	++	+		Eve	+	+++
**Ventral thalamus**			LVPO	+	+		SuVe	+	+
Rt	+	+	SPO	+	+/−		LVe	+	+
ZI	+	++	LSO	+	+		VeCb	−	+/−
STh	+	+	PL/ILL/VLL	+	++		SpVe	−	+/−
**Epithalamus**			tz	−	+		X	+	+
LHb	+/−	+/−	Tz	++	+		Others		
MHb	++++	−	DC	+	+		A5	++	−
fr	++++	−	GrC	+	+		Bar	++	−
			VCA	+	+		CGA	++	+/−
			VCP	+	++		CGPn	+	+
			SGI	+	+/−		Cu	−	+
			Cranial nerves				Ecu	−	+++
			6N	+	+		IO	+	−
			7N/P7	+/++	+		LC	+	−
			10N	−	+		Pn	++	+++
			Amb	+++	+/−		PP	+	+
			12N	+/−	++		Sol	+	+/−

Expression: *−* not detectable, *±* weak, *+* moderate, *++* dense, *+++* very dense

**Fig. 4:**
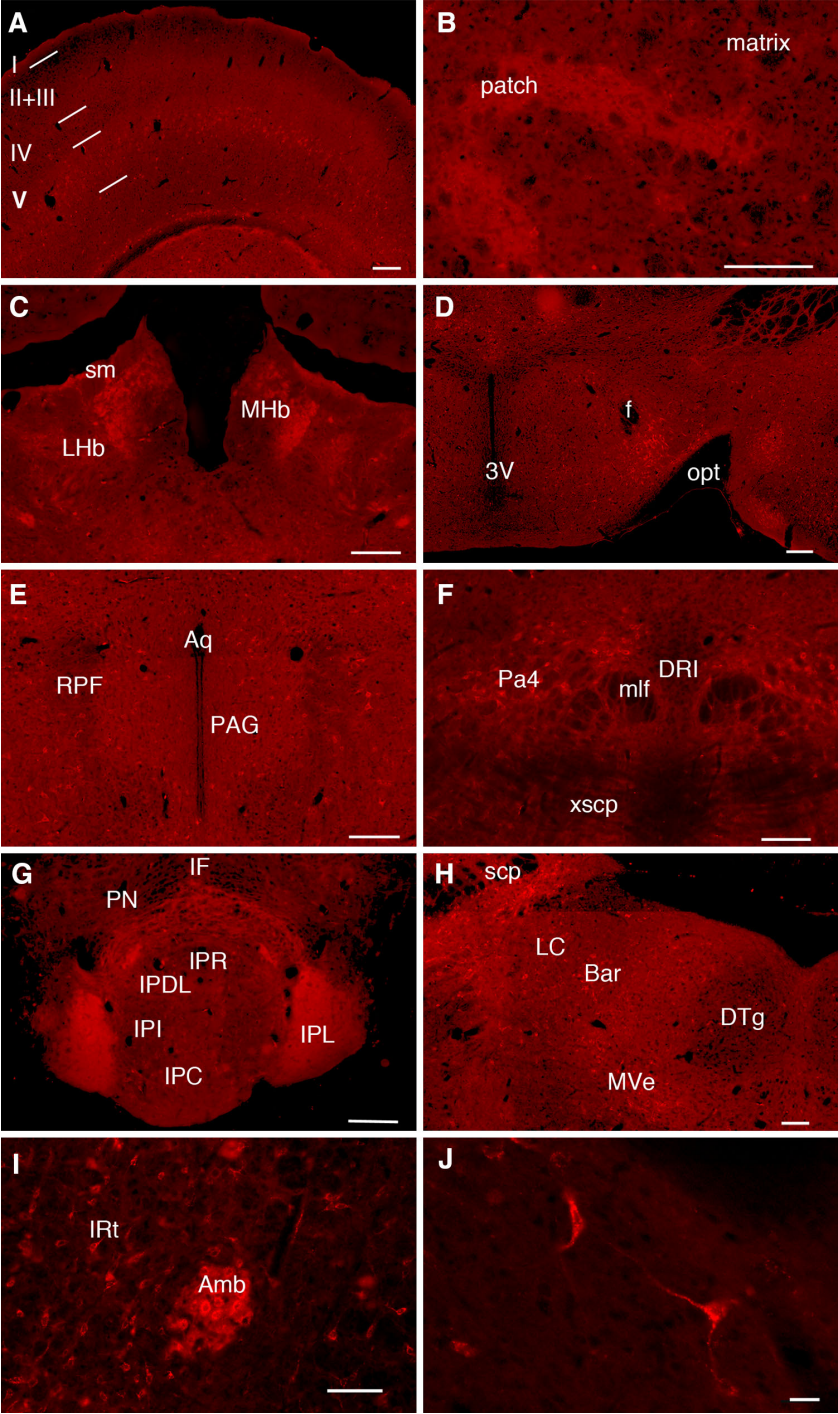
Brain mapping of MOR-mcherry expression at cellular resolution MOR-mcherry expression is observed in discrete neuronal populations at the level of the **a** cortex, **b** striatum, **c** habenula, **d** lateral hypothalamus, **e** periaqueductal gray matter, **f** paratrochlear nucleus, **g** interpeduncular nucleus, **h** locus coeruleus area, **i** nucleus ambiguus, **j** detail of the lateral hypothalamus. *Scale bars* 200 and 20 μm (**j**)

Both MOR-mcherry and DOR-eGFP distributions in the brain are in full agreement with the previous reports in mice and rats based on ligand binding (Slowe et al. 1999; Lesscher et al. 2003; Kitchen et al. 1997; Goody et al. 2002), GTPγS incorporation (Tempel and Zukin 1987; Pradhan and Clarke 2005) or mRNA detection (Mansour et al. 1995; George et al. 1994; Cahill et al. 2001) (for a review see (Le Merrer et al. 2009)). In addition, we detected MOR-mcherry expression in discrete groups of neurons (Fig. 4; online atlas) that could not be previously resolved using autoradiography or in situ hybridization. Therefore, the approach further refines our current knowledge of MOR distribution.

In the spinal cord, localization of the fluorescent signals associated with MOR-mcherry and (Mansour et al. 1987) DOR-eGFP is also in agreement with mRNA distribution, radioligand binding and immunohistochemical data collected in rat (Wang et al. 2010; Trafton et al. 2000; Gray et al. 2006) and mice (Scherrer et al. 2009). The fluorescence associated with MOR-mcherry was predominantly present in the superficial layers of the dorsal horn, mainly lamina II, but somas could be detected in all layers (online atlas).

In DRGs, DOR-eGFP was present in neurons with small-, medium-, and large-diameter somata with predominance in the latter, consistent with enrichment in myelinated afferents as previously reported (Scherrer et al. 2009). MOR-mcherry was also expressed in the three types of neurons but was most abundant in DRG neurons with small diameter cell in agreement with previous immunohistochemical detection in rats and mice (Scherrer et al. 2009; Wang et al. 2010; Rau et al. 2005).

Altogether, both MOR-mcherry and DOR-eGFP fluorescent signals are consistent with currently available data from the literature. This designates the double fluorescent knock-in mouse as a unique tool to fine map receptor expression at subcellular level, and addresses brain sites of receptor co-expression.

### Neuroanatomy of MOR-mcherry/DOR-eGFP neurons in double mutant mice

**Fig. 5:**
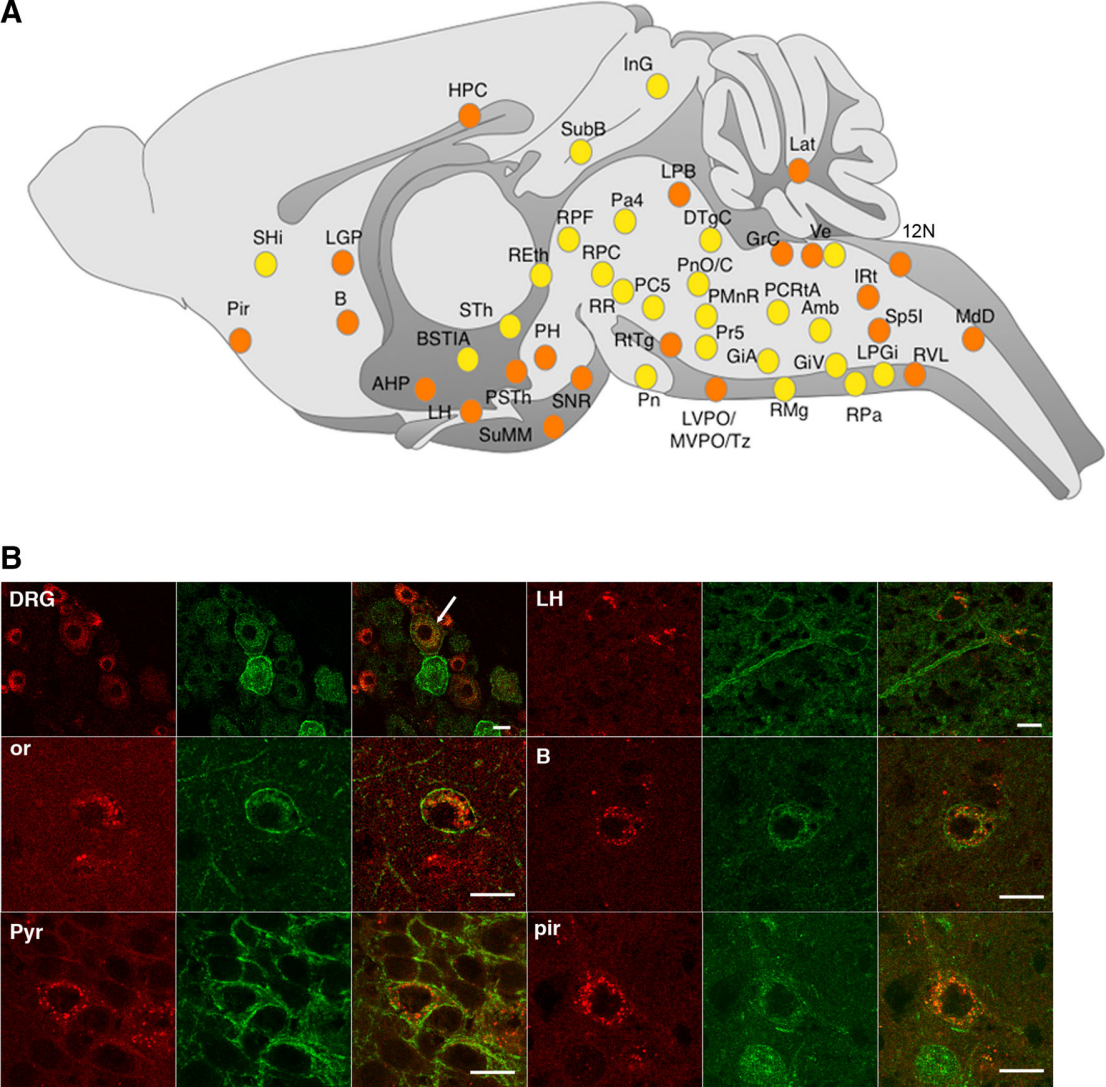
MOR/DOR neurons in the nervous system. **a** Brain mapping of neurons co-expressing MOR-mcherry and DOR-eGFP under basal conditions (*orange filled circle*) or following treatment with the DOR agonist SNC 80 (10 mg/kg, s.c. for 2 h) (*yellow filled circle*). See list for abbreviations. **b** Co-localization of MOR-mcherry and DOR-eGFP within the same neuron in dorsal root ganglia (DRG) (*white arrow*), oriens (or) and pyramidal (pyr) layers of the hippocampus, lateral hypothalamus (LH), basal nucleus of Meynert (B), piriform cortex (pir). *Scale bars* 10 μm

Under basal conditions, we identified a limited number of regions in which both MOR-mcherry and DOR-eGFP fluorescent proteins could be detected in the same neuron (Fig. 5a, b; online atlas). Regions with most significant co-expression were the hippocampus, the hypothalamus, the lateral parabrachial nucleus and vestibular nuclei. Additional regions included the piriform cortex, the auditory pathway, as well as regions involved in the control of movement and posture or relaying somatosensory or motor information to the autonomic nervous system. Cellular co-expression of MOR-mcherry and DOR-eGFP in the lateral hypothalamus and the rostroventral medulla is consistent with previous identification, respectively, by a combination of electrophysiology and immunohistochemistry (Pedersen et al. 2011). Also, cellular co-expression of MOR-mcherry and DOR-eGFP in the main nucleus of the trapezoid body, the rostroventrolateral medulla, the hippocampus, the pons and the hypothalamus is in agreement with MOR–DOR detection using heteromer-specific antibodies (Gupta et al. 2010).

**Fig. 6:**
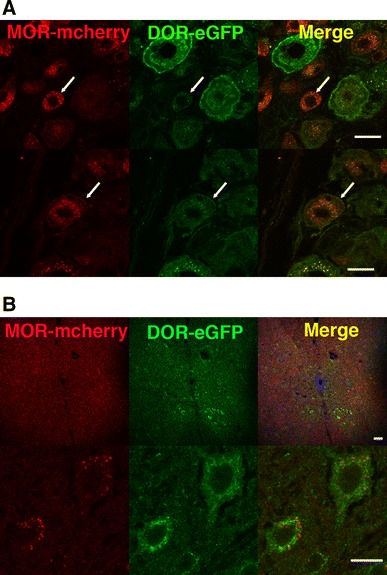
MOR and DOR neuronal co-expression in dorsal root ganglia and spinal cord. **a** In dorsal root ganglia, MOR-mcherry and DOR-eGFP are co-expressed under basal conditions in small- and medium-size neurons in addition to large neurons (shown in Fig. 5b) (*arrows*). *Scale bars* 20 μm. **b** Neurons co-expressing MOR-mcherry and DOR-eGFP are visualized in the different layers of the spinal cord following treatment with the delta agonist SNC 80 (10 mg/kg, s.c., 2 h). General view (*top panel*) and individual neurons (*bottom panel*). *Scale bars* 10 μm

In the literature, DRGs represent one of the few sites where MOR/DOR co-localization was studied (Scherrer et al. 2009; Wang et al. 2010; Rau et al. 2005). Here, we found that MOR-mcherry and DOR-eGFP co-expression was restricted to discrete populations of small-, medium-, and large-size DRG neurons (Fig. 6a). We estimate that about 40 % of DOR-eGFP-positive (43 ± 8 %, *n* = 20) and one-third of MOR-mcherry-positive (35 ± 5 %, *n* = 20) neurons express the two receptors. Large neurons represent about 37 ± 8 % (*n* = 20) of the total number of neurons co-expressing the two receptors. The extent of receptor co-expression in this study is larger than previously reported using native MOR immunodetection in DOR-eGFP knock-in mice (Scherrer et al. 2009) but remains consistent with MOR and DOR being predominantly expressed on distinct populations of somatosensory neurons.

**Fig. 7:**
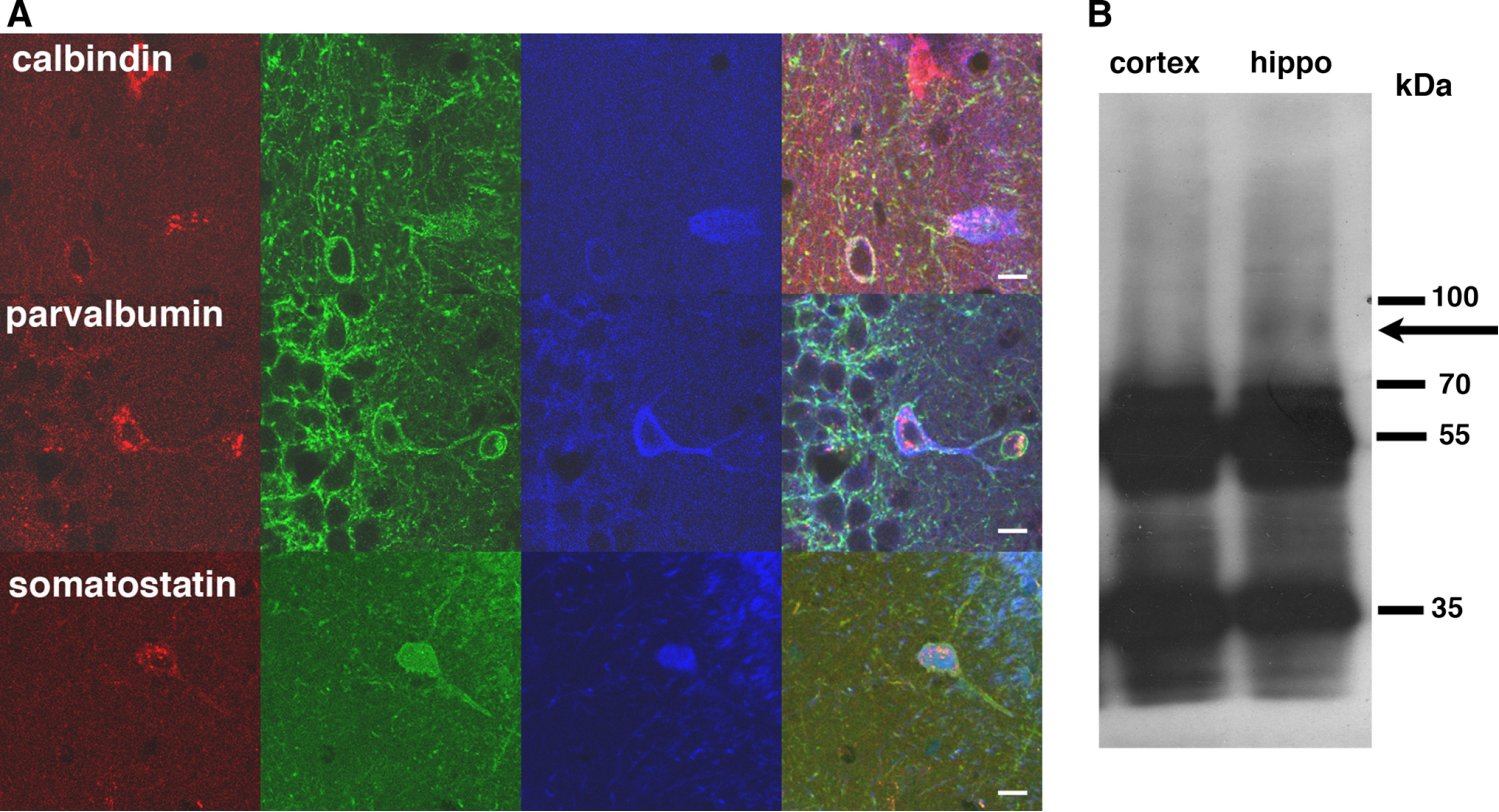
Fine mapping of MOR/DOR neurons in the hippocampus. **a** Neurons co-expressing MOR-mcherry and DOR-eGFP are identified by co-localization with the neuronal markers calbindin, parvalbumin or somatostatin. *Scale bars* 10 μm. **b** MOR and DOR form heteromers in the hippocampus. Immunoprecipitation with rabbit polyclonal anti-mcherry antibodies was performed on solubilized membranes from the cortex (cx) or hippocampus (hippo). Western blotting of the isolated immunocomplexes using rabbit polyclonal anti eGFP antibodies detected the DOR-eGFP construct (*arrow*) in the hippocampus where MOR-mcherry and DOR-eGFP co-localize

Using DOR-eGFP mice, we previously identified neuronal populations expressing DOR in the hippocampus (Erbs et al. 2012). Here, co-localization of MOR-mcherry and DOR-eGFP fluorescent signals was observed in GABAergic interneurons that control the firing rate of glutamatergic neurons (Fig. 7a). Co-expression in parvalbumin-positive neurons from the pyramidal layer suggests that these neurons are basket or chandelier cells (Erbs et al. 2012). Co-expression is also observed in horizontal somatostatin-positive cells close to the alveus which points to oriens-lacunosum moleculare or hippocampo-septal neurons (Erbs et al. 2012).

We then investigated whether MOR/DOR heteromers are detectable in the hippocampus, where extensive co-localization was observed. Co-immunoprecipitation experiments using antibodies directed against the fluorescent proteins indeed revealed close physical proximity between the two receptors supporting the hypothesis that MOR/DOR heteromers can exist in this structure (Fig. 7b).

### Neuroanatomy of MOR-mcherry/DOR-eGFP neurons in double mutant mice following treatment with SNC 80

To increase detection of DOR-eGFP expressing cell bodies, mice were treated with the DOR-selective agonist SNC 80 (10 mg/kg) for 2 h before perfusion. This drug induces DOR internalization in vivo, and subsequent degradation in lysosomal compartments (Pradhan et al. 2009), leading to concentrate DOR-eGFP fluorescence in the soma while depleting DOR-eGFP staining from neuron terminals. Upon treatment, the number of detected DOR-eGFP cell bodies increased substantially, revealing about twice as many regions with MOR-mcherry/DOR-eGFP co-expressing neurons compared to basal conditions (Fig. 5a). Most of these new areas concentrate in the brainstem and midbrain and are associated with distinct functional sensorimotor pathways (see “Discussion”). SNC80 treatment also revealed co-expression of the two receptors in neurons distributed across all layers of the spinal cord (Fig. 6b). This latter observation is concordant with a previous study reporting physical MOR/DOR interaction in the spinal cord using co-immunoprecipitation experiments (Gomes et al. 2004).

Notably, SNC80 treatment did not reveal any co-localization in the telencephalon other than the piriform cortex already identified under basal conditions. In essence, whether or not DOR agonist treatment was used, we could not detect MOR/DOR co-expression in brain areas where opioid receptors are most extensively studied. This includes the cortex, as well as ventral and dorsal striatum, where receptor cross talk may not be a major operating mechanism. A previous study using ELISA with heteromer-specific antibodies nevertheless reported MOR/DOR co-expression in these regions (Gupta et al. 2010), a discrepancy that could result from very low levels of co-localized receptors undetectable in our approach.

In conclusion, red/green mapping in double mutant mouse submitted to DOR agonist treatment confirms that double-positive MOR/DOR neurons are essentially distributed in midbrain and hindbrain, whereas neurons expressing a single receptor seem mostly restricted to the forebrain. Mining the MOR/DOR atlas, therefore, led us to postulate that functional interactions between MOR and DOR may operate at cellular level mainly in neurons forming midbrain and hindbrain pathways (“Discussion”).

## Discussion

Functional interactions between GPCRs via signaling cross talk or heteromerization have long been established in vitro, but the relevance of these mechanisms in vivo is the subject of intense investigation. In this debate, opioid receptors are leading candidates because mechanisms underlying functional interactions across opioid receptors have important implications for opioid physiology and therapy. MOR/DOR interactions in cellular models are well established. However, there is very little evidence to support in vivo co-expression of MOR and DOR within the same neuron, a prerequisite for either signaling cross talk or physical interactions. Using double fluorescent knock-in mice expressing functional MOR and DOR in fusion with eGFP and mcherry, respectively, we produced a MOR/DOR brain atlas (http://mordor.ics-mci.fr/ ). This searchable database shows fine mapping of the two receptors throughout the nervous system with cellular resolution, and allows the identification of MOR/DOR neurons co-expressing the two receptors in vivo.

To get further insights into the molecular bases of receptor co-expression, we also investigated potential MOR/DOR physical interactions in the hippocampus where extensive co-localization is observed under basal conditions. Our co-immunoprecipitation experiments indicated MOR/DOR physical proximity in this structure, and in-depth analysis is now required to extend this finding to other regions showing MOR/DOR neuronal co-localization.

### Methodological considerations

Detection of MOR/DOR neuronal co-expression relies on the concomitant visualization of the two fluorescent signals but also on our ability to fit their distribution with identifiable neuronal structures. In most brain regions, DOR and MOR expression levels range from 10 to 60 fmol/mg (Slowe et al. 1999; Lesscher et al. 2003; Kitchen et al. 1997; Goody et al. 2002), which raises the possibility of overlooking areas with the lowest expression levels. However, DOR and MOR fluorescent constructs were detected in all regions with previously identified wild-type DOR or MOR expression. Indeed, MOR-mcherry was readily observed owing to the intracellular accumulation of the red fluorescence even in low expressing neurons. Accordingly, subsequent amplification with mcherry specific antibodies did not significantly improve MOR-mcherry detection. On the contrary, the green fluorescence associated with DOR was often weak, which required amplification with eGFP-specific antibodies for proper visualization. In addition, DOR-eGFP did not accumulate in the soma as for MOR-mcherry and the green fluorescence was often associated with passing fibers. Therefore, identification of neuronal cell bodies was hampered because of the lack of visual landmarks in particular in structures where the tissue organization was very dense. This limitation was overcome by treating animals with the DOR agonist SNC 80 that concentrated the green fluorescence in the soma in a manner similar to MOR-mcherry. Combining fluorescence amplification with agonist treatment significantly enhanced the sensitivity of our approach and drastically improved identification of DOR-eGFP neurons and, hence, MOR/DOR neurons (Fig. 5a). Nonetheless, the latter may still have escaped detection in regions of very low expression.

Analyzing MOR/DOR co-localization throughout the entire brain brought interrogations about the possible implications of neuronal co-expression. To address MOR/DOR functional role, we identified specific neuronal circuits in which neurons co-expressing the two receptors were located. For this purpose, networks were built that encompass regions of MOR/DOR co-localization with previously documented anatomical connections and behavioral outcome. Though this approach remains speculative and needs experimental validation, it offers a novel frame to investigate the in vivo implications of MOR/DOR co-expression.

### Eating and sexual behaviors

A most remarkable observation from the MOR/DOR atlas is the widespread distribution of MOR/DOR co-expressing neurons in the brainstem. Receptor co-expression may reflect ancestral expression that was preserved across evolution owing to successful contribution to survival. Opioid receptors have been identified throughout vertebrates including frogs and fishes, and their expression likely results from initial duplication of an ancestral single MOR/DOR gene (Stevens 2009). MOR and DOR may therefore cooperate within neurons to regulate primitive aspects of animal behavior such as sensitivity to somatosensory stimuli and subsequent motor reflexes. Notably, modifications in binding and signaling properties observed in heterologous systems suggest that MOR/DOR co-expression enhances opioid-induced inhibition of neuronal activity (Rozenfeld and Devi 2011). Endogenous opioid peptides, therefore, may efficiently control primal behaviors through additive, synergistic or other mechanisms that differ from activation of a single receptor.

Another striking feature is the lack of MOR/DOR neurons in the telencephalon with the notable exception of the piriform cortex that integrates odorant stimuli (Wilson and Sullivan 2011). This may reflect the importance of odorant stimuli as key sensory inputs contributing to food search and recognition, identification of sexual partners or predator avoidance.

**Fig. 8:**
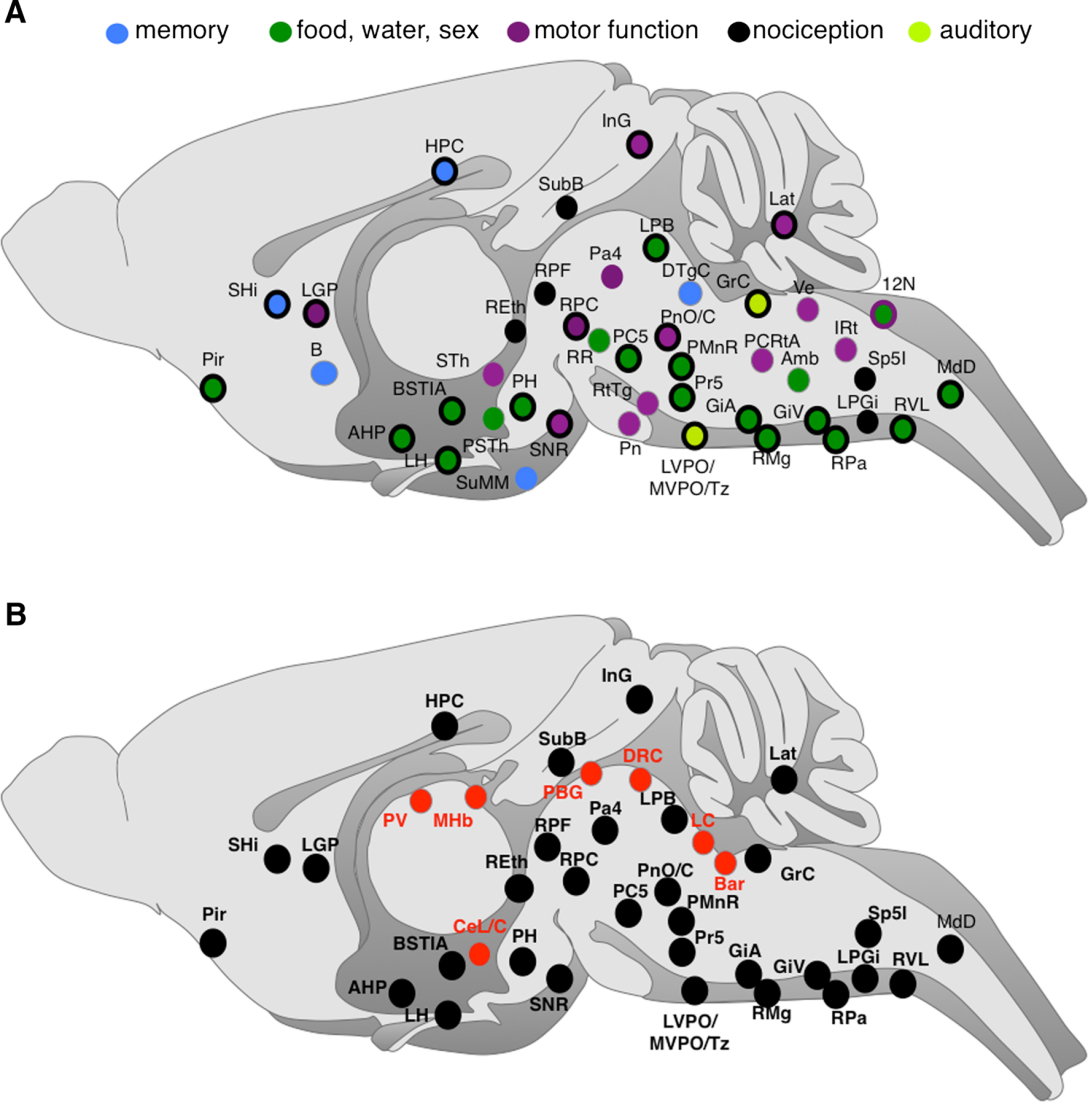
MOR/DOR neurons concentrate in networks essential for survival. **a** MOR/DOR co-expressing neurons are detected in pathways essential for survival. Areas belonging to pathways classically related to memory (*filled blue circle*), sex and food and water consumption (*filled dark green*), motor function (*filled purple circle*), nociception (*filled black circle*) and audition (*filled light green circle*) are indicated. Regions belonging to two networks are presented as a two-color circle. **b** In addition to MOR/DOR-containing neurons (*filled black circle*), brain regions activated by painful stimuli also included neurons expressing MOR only (*filled red circle*) See list for abbreviations

Neurons co-expressing MOR and DOR are detected in neuronal circuits that process food intake (Reis 2007; Shin et al. 2011; De Luca et al. 2007), NaCl and water uptake (Shin et al. 2011) or regulation of the sexual activity in both males (Hamson and Watson 2004) and females (Komisaruk and Whipple 2005). These include the intramygdaloid part of the bed nucleus of the stria terminalis, the anterior, lateral and posterior hypothalamus, the hippocampus, and the lateral parabrachial nucleus (Fig. 8a). Noteworthy, MOR/DOR neurons in the lateral hypothalamus overlap with orexin-positive neurons and may modulate the orexigenic component of eating behavior by exerting inhibitory controls (Harris and Aston-Jones 2006). Also MOR/DOR neuronal co-expression is found in amygdaloid areas, where lesions produce weight gain and obesity in female rats (King et al. 2003).

Taken together, MOR/DOR cross talk at cellular level may operate in neural circuits involved in behaviors associated with body homeostasis and sexual activities.

### Perception and processing of aversive stimuli

Another fascinating observation is the presence of MOR/DOR neurons in subcortical networks responding to presentation of noxious or non-noxious aversive stimuli (Fig. 8a), as described in a recent translational study, that integrates functional neuroanatomy in rodents and meta analysis of PET and fMRI data in humans (Hayes and Northoff 2011, 2012). Subcortical areas involved in these core aversion-related networks that also show MOR/DOR co-localization include the hippocampus, hypothalamus, nuclei from the midbrain such as the red and pontine reticular nuclei, several areas from the pons including the parabrachial nucleus and the rostral ventral medulla.

Regarding pain processing, co-expression of the two receptors is high throughout nociceptive pathways, including the rostral ventral medulla, lateral parabrachial nucleus, spinal cord and DRGs (Fig. 8b). Interestingly, we were not able to identify concomitant MOR and DOR expression in neurons of the periaqueductal gray, a brain area central to pain control (Basbaum et al. 2009). MOR and DOR may function independently at this level of pain processing, although we cannot exclude that fluorescent signals remained below detection thresholds.

Finally, MOR/DOR neuronal co-expression is also observed in the memory network involving the hippocampus, some septal areas and mammillary bodies (Fig. 8a). MOR/DOR interactions may therefore modulate hippocampal activity and, in particular, the CA1 area operating as a coincidence detector (Faget et al. 2012; Duncan et al. 2012). Similarly, the detected MOR/DOR neurons may influence odor processing in the piriform cortex considered as another coincidence detector (Wilson and Sullivan 2011).

In conclusion, endogenous opioid peptides may trigger MOR/DOR-specific responses in brain pathways contributing to avoid and/or cope with threatening situations. Interestingly, networks associated to perception of aversive painful stimuli also encompass areas where MOR is detected alone. In these regions, presented in Fig. 8b, MOR-mediated control is likely the predominant mechanism.

### Sensorimotor pathways

The surprising observation of MOR/DOR co-expression in brain areas involved in motor activity (Fig. 8a) expands our current understanding of opioid physiology. Food, water or salt intake require mandibular movements involved in masticatory reflexes and jaw movements, where the two receptors may co-modulate somatomotor orofacial activity and influence parasympathetic responses and cardiovascular function associated with feeding (Goto and Swanson 2004; Dong and Swanson 2003; Mascaro et al. 2009). Accordingly, MOR/DOR neurons are distributed throughout descending projections to hindbrain preganglionic parasympathetic nuclei and orofacial motor pattern generators. We also observed MOR/DOR neurons in brainstem networks associated to motor aspects of sexual activity, including the (para) gigantocellular reticular formation and lateral vestibular nucleus that participate to the regulation of penile reflexes (Hamson and Watson 2004).

Detection of aversive stimuli, need for food, water or sexual attraction also requires appropriate motor responses. The strong link between aversion-related networks and the autonomic nervous system is especially remarkable and suggests that MOR/DOR neuronal co-expression is essential for the modulation of both ascending somatosensory information and corresponding descending reflex responses intended to protect the individual. Accordingly, MOR/DOR neurons are present in areas of the basal ganglia contributing to integration of information that control motor responses to auditory, visual or olfactory stimuli (Fig. 8a). In addition, we observed MOR/DOR neurons in the auditory complex and the vestibular system that participate in body balance (Sturnieks et al. 2008) (Fig. 8a). Finally, MOR/DOR neuronal co-expression in the auditory system may modulate the processing of auditory inputs and hence impact on male copulation through strong connections to the (para)gigantocellular reticular formations (Bellintani-Guardia et al. 1996).

### Therapeutic implications

Targeting MOR/DOR-mediated signaling mechanisms, which would be distinct from single receptor signaling, may lead to develop innovative therapeutic approaches. The identification of neural networks with potential intracellular MOR/DOR interactions provides valuable hints toward selected therapeutic effects.

Mu opioid receptors represent a major target for analgesics, but progressive loss in opioid drug efficacy constitutes a key challenge for clinicians. On the other hand, the notion that DORs significantly contribute to the development of morphine tolerance has often been put forward, but the molecular mechanisms of this particular MOR/DOR interaction remain elusive (Cahill et al. 2007). Specific trafficking and signaling properties of MOR/DOR heteromers were reported in heterologous systems, and the therapeutic potential of receptor heteromers is being considered to reduce opioid tolerance (Berger and Whistler 2011; Gomes et al. 2013). The high co-localization of MOR and DOR in neurons from nociceptive pathways supports this view and designates the putative heteromers as an attractive target in pain management. In addition, the absence of neuronal co-localization in the pre-Bötzinger complex suggests that MOR/DOR specific targeting may produce analgesic effects devoid of respiratory depression side effects.

Neurons co-expressing the two receptors are also abundant in brainstem nuclei tightly connected with the autonomic nervous system. At these sites, the two receptors may functionally cooperate in the generation of somatic and autonomic symptoms during drug withdrawal. The rostral ventromedial medulla, especially the raphe magnus and nucleus paragigantocellularis are engaged in the expression of several aspects of physical opioid withdrawal, via their efferent projections to autonomic and somatic motor neurons. In addition, the nucleus paragigantocellularis represents the major source of excitatory drive to the locus coeruleus during withdrawal (Williams et al. 2001). Targeting MOR/DOR heteromers may therefore represent an attractive strategy to reduce opioid withdrawal, and possibly withdrawal signs associated with other drugs of abuse. As such, MOR/DOR heteromers have been proposed as a promising molecular entity for the development of selective antagonists to treat alcoholism (van Rijn and Whistler 2009).

Finally, neuronal co-expression of the two receptors in the lateral hypothalamus has interesting implications. MOR/DOR co-localization occurs partly in orexin-positive neurons. Hence, receptor heteromers may represent a potential target for novel strategies to treat obesity. Also, orexin-positive neurons are critical for both food and drug reward. MOR/DOR-specific mechanisms may therefore be targeted to reduce drug-seeking behavior (Aston-Jones et al. 2009).

## Conclusion

Overall, MOR/DOR co-expressing neurons are extremely scarce in forebrain networks responsible for higher-order processing, and are detectable mainly at the level of mid- and hindbrain regions with connections to the autonomic nervous system. Mining the brain atlas therefore suggests that functional interactions between MOR and DOR operate predominantly at circuitry level for mood control, reward processing and cognition, whereas the two receptors may cooperate intracellularly in neural networks essential for survival.

Close physical proximity strongly supports the existence of in vivo mu–delta heteromers in the hippocampus. MOR/DOR physical association in neural networks associated with abnormal nociception, aversive aspects of drug withdrawal or eating disorders represents an attractive option for drug design. The identification of neurons co-expressing the two receptors in the nervous system will now initiate in-depth in vivo investigations to understand molecular mechanisms underlying MOR/DOR cooperativity in selected neural networks, and their functional significance in complex behaviors. Ultimately, the MOR/DOR atlas resource provides a proof-of-principle approach to address the challenging issue of GPCR interactions and heteromerization in physiology and disease.

## Electronic supplementary material

Below is the link to the electronic supplementary material.

Supplementary material 1 (PDF 632 kb)

## Abbreviations

3N: Oculomotor nucleus
4N: Trochlear nucleus
6N: Abducens nucleus
7N: Facial nucleus
10N: Dorsal motor nucleus of vagus
12N: Hypoglossal nucleus
3V: Third ventricle
A5: A5 noradrenaline cells
AAV: Anterior amygdaloid area, ventral part
AcbC: Accumbens nucleus, core
AcbS: Accumbens nucleus, shell
ACo: Anterior cortical amygdaloid nucleus
AD: Anterodorsal thalamic nucleus
AHA: Anterior hypothalamic area, anterior part
AHC: Anterior hypothalamic area, central part
AHP: Anterior hypothalamic area, posterior part
AHi: Amygdalohippocampal area
AI: Agranular insular cortex
alv: alveus
AM: Anteromedial thalamic nucleus
Amb: Ambiguus nucleus
AOE: Anterior olfactory nucleus, external part
AVPe: Anteroventral periventricular nucleus
APir: Amygdalopiriform transition area
APT: Anterior pretectal nucleus
Aq: Aqueduc (Sylvius)
Arc: Arcuate hypothalamic nucleus
AStr: Amygdalostriatal transition area
Atg: Anterior tegmental nucleus
aud: Auditory cortex
AVDM: Anteroventral thalamic nucleus, dorsomedial part
AVVL: Anteroventral thalamic nucleus, ventrolateral part
B (Meynert): Basal nucleus
BAOT: Bed nucleus of the accessory olfactory tract
Bar: Barrington’s nucleus
BIC: Nucleus of the brachium of the inferior colliculus
BLA: Basolateral amygdaloid nucleus, anterior part
BLP: Basolateral amygdaloid nucleus, posterior part
BMA: Basomedial amygdaloid nucleus, anterior part
BMP: Basomedial amygdaloid nucleus, posterior part
BSTIA: Bed nucleus of the stria terminalis, intraamygdaloid division
BSTLD: Bed nucleus of the stria terminalis, lateral division, dorsal part
BSTLI: Bed nucleus of the stria terminalis, lateral division, intermediate part
BSTLP: Bed nucleus of the stria terminalis, lateral division, posterior part
BSTLV: Bed nucleus of the stria terminalis, lateral division, ventral part
BSTMA: Bed nucleus of the stria terminalis, medial division, anterior part
BSTMP: Bed nucleus of the stria terminalis, medial division, posterior part
BSTMV: Bed nucleus of the stria terminalis, medial division, ventral part
CA1: Field CA1 of hippocampus
CA3: Field CA3 of hippocampus
CbCx: Cerebellar cortex
cCM: Central medial thalamic nucleus, caudal part
CeC: Central amygdaloid nucleus, capsular part
CeI: Central amygdaloid nucleus, intermediate part
CeL: Central amygdaloid nucleus, lateral division
CeM: Central amygdaloid nucleus, medial division
Cg: Cingulate cortex
CGA: Central gray, alpha part
CGPn: Central gray of the pons
CIC: Central nucleus of the inferior colliculus
Cl: Claustrum
CL: Centrolateral thalamic nucleus
CN: Cochlear nuclei
CnF: Cuneiform nucleus
CPO: Caudal periolivary nucleus
CPu: Caudate putamen
Cu: Cuneate nucleus
CVL: Caudoventrolateral reticular nucleus
CxA: Cortex-amygdala transition zone
DC: Dorsal cochlear nucleus
DCIC: Dorsal cortex of the inferior colliculus
Den: Dorsal endopiriform nucleus
df: Dorsal fornix
DG: Dentate gyrus
DI: Dysgranular insular cortex
Dk: Nucleus of Darkschewitsch
DLG: Dorsal lateral geniculate nucleus
DLPAG: Dorsolateral periaqueductal gray
DM: Dorsomedial hypothalamic nucleus
DMPAG: Dorsomedial periaqueductal gray
DMTg: Dorsomedial tegmental area
DP: Dorsal peduncular cortex
DpG: Deep gray layer of the superior colliculus
DPGi: Dorsal paragigantocellular nucleus
DpMe: Deep mesencephalic nucleus
DPO: Dorsal periolivaly region
DRC: Dorsal raphe nucleus, caudal part
DRD: Dorsal raphe nucleus, dorsal part
DRI: Dorsal raphe nucleus, interfascicular part
DRV: Dorsal raphe nucleus, ventral part
DTg: Dorsal tegmental nucleus
DTgC: Dorsal tegmental nucleus, central part
DTgP: Dorsal tegmental nucleus, pericentral part
DTT: Dorsal tenia tecta
ECIC: External cortex of the inferior colliculus
Ect: Ectorhinal cortex
Ecu: External cuneate nucleus
Epl: External plexiform layer of the olfactory bulb
EPlA: External plexiform layer of the accessory olfactory bulb
Eth: Ethmoid thalamic nucleus
EVe: Nucleus of origin of efferents of the vestibular nerve
EW: Edinger-Westphal nucleus
fi: Fimbria of the hippocampus
fr: Fasciculus retroflexus
FrA: Frontal association cortex
GI: Granular insular cortex
Gi: Gigantocellular reticular nucleus
GiA: Gigantocellular reticular nucleus, alpha part
GiV: Gigantocellular reticular nucleus, ventral part
Gl: Glomerular layer of the olfactory bulb
GrA: Granule cell layer of the accessory olfactory bulb
GrC: Granular layer of the cochlear nuclei
GrO: Granule cell layer of the olfactory bulb
HDB: Nucleus of the horizontal limb of the diagonal band
I: Intercalated nuclei of the amygdala
I5: Intertrigeminal nucleus
IAD: Interanterodorsal thalamic nucleus
IAM: Interanteromedial thalamic nucleus
ICjM: Islands of Caleja, major island
IF: Interfascicular nucleus
IGL: Intergeniculate leaflets
IL: Infralimbic cortex
ILL: Intermediate nucleus of the lateral lemniscus
IMD: Intermediodorsal thalamic nucleus
InC: Interstitial nucleus of Cajal
InG: Intermediate gray layer of the superior colliculus
IntP: Interposed cerebellar nucleus, posterior part
InWh: Intermediate white layer of the superior colliculus
IO: Inferior olive
IPAC: Interstitial nucleus of the posterior limb of the anterior commissure
IPC: Interpeduncular nucleus, caudal subnucleus
IPDL: Interpeduncular nucleus, dorsolateral subnucleus
IPDM: Interpeduncular nucleus, dorsomedial subnucleus
IPI: Interpeduncular nucleus, intermediate subnucleus
IPl: Internal plexiform layer of the olfactory bulb
IPL: Interpeduncular nucleus, lateral subnucleus
IPR: Interpeduncular nucleus, rostral subnucleus
IPRL: Interpeduncular nucleus, rostral subnucleus, lateral part
IRt: Intermediate reticular nucleus
LA: Lateroanterior hypothalamic nucleus
Lat: Lateral (dentate) cerebellar nucleus
LC: Locus coeruleus
LDDM: Laterodorsal thalamic nucleus, dorsomedial part
LDMM: Laterodorsal thalamic nucleus, dorsomedial part
LDTg: Laterodorsal tegmental nucleus
LDVL: Laterodorsal thalamic nucleus, ventrolateral part
LEnt: Lateral entorhinal cortex
LGP: Lateral globus pallidus
LH: Lateral hypothalamic area
LHb: Lateral habenular nucleus
LM: Lateral mammillary nucleus
LOT: Nucleus of the lateral olfactory tract
LPAG: Lateral periaqueductal gray
LPBC: Lateral parabrachial nucleus, central part
LPBD: Lateral parabrachial nucleus, dorsal part
LPBE: Lateral parabrachial nucleus, external part
LPBI: Lateral parabrachial nucleus, internal part
LPBS: Lateral parabrachial nucleus, superior part
LPBV: Lateral parabrachial nucleus, ventral part
LPGi: Lateral paragigantocellular nucleus
LPO: Lateral preoptic area
LRt: Lateral reticular nucleus
LSD: Lateral septal nucleus, dorsal part
LSI: Lateral septal nucleus, intermediate part
LSO: Lateral superior olive
LSV: Lateral septal nucleus, ventral part
LVe: Lateral vestibular nucleus
LVPO: Lateroventral periolivary nucleus
M1: Primary motor cortex
M2: Secondary motor cortex
MA3: Medial accessory oculomotor nucleus
mcp: Middle cerebellar peduncle
MCPO: Magnocellular preoptic nucleus
MD: Mediodorsal thalamic nucleus
MDC: Mediodorsal thalamic nucleus, central part
MdD: Medullary reticular nucleus, dorsal part
MDL: Mediodorsal thalamic nucleus, lateral part
MDM: Mediodorsal thalamic nucleus, medial part
ME: Median eminence
Me5: Mesencephalic trigeminal nucleus
MeAD: Medial amygdaloid nucleus, anterior dorsal
MeAV: Medial amygdaloid nucleus, anteroventral part
Med: Medial (fastigial) cerebellar nucleus
MEnt: Medial entorhinal cortex
MePD: Medial amygdaloid nucleus, posterodorsal part
MePV: Medial amygdaloid nucleus, posteroventral part
MGD: Medial geniculate nucleus, dorsal part
MGM: Medial geniculate nucleus, medial part
MGP: Medial globus pallidus (entopeduncular nucleus)
MGV: Medial geniculate nucleus, ventral part
MHb: Medial habenular nucleus
Mi: Mitral cell layer of the olfactory bulb
MiA: Mitral cell layer of the accessory olfactory bulb
MiTg: Microcellular tegmental nucleus
ML: Medial mammillary nucleus, lateral part
mlf: Medial longitudinal fasciculus
MM: Medial mammillary nucleus, medial part
MMn: Medial mammillary nucleus,median part
MnPO: Median preoptic nucleus
MnR: Median raphe nucleus
Mo5: Motor trigeminal nucleus
MPA: Medial preoptic area
MPB: Medial parabrachial nucleus
MPBE: Medial parabrachial nucleus, external part
MPOL: Medial preoptic nucleus, lateral part
MPOM: Medial preoptic nucleus, medial part
MPT: Medial pretectal nucleus
MS (Ld): Medial septal nucleus
MTu: Medial tuberal nucleus
Mve: Medial vestibular nucleus
MVeMC: Medial vestibular nucleus, magnocellular part
MVePC: Medial vestibular nucleus, parvicellular part
MVPO: Medioventral periolivary nucleus
Op: Optic nerve layer of the superior colliculus
OPC: Oval paracentral thalamic nucleus
opt: Optic tract
OPT: Olivary pretectal nucleus
P7: Perifacial zone
Pa4: Parathrochlear nucleus
PaAP: Paraventricular hypothalamic nucleus, anterior parvicellular part
PAG: Periaqueductal gray
PaLM: Paraventricular hypothalamic nucleus, lateral magnocellular part
PaS: Parasubiculum
PaV: Paraventricular hypothalamic nucleus, ventral part
PBG: Parabigeminal nucleus
PC: Paracentral thalamic nucleus
PC5: Parvicellular motor trigeminal nucleus
PCRt: Parvicellular reticular nucleus
PCRtA: Parvicellular reticular nucleus, alpha part
PDTg: Posterodorsal tegmental nucleus
Pe: Periventricular hypothalamic nucleus
PeF: Perifornical nucleus
PF: Parafascicular thalamic nucleus
PH: Posterior hypothalamic area
PIL: Posterior intralaminar nucleus
Pir: Piriform cortex
PL: Paralemniscal nucleus
PLCo: Posterolateral cortical amygdaloid nucleus
PLi: Posterior limitans thalamic nucleus
PMCo: Posteromedial cortical amygdaloid nucleus
PMD: Premammillary nucleus, dorsal part
PMnR: Paramedian raphe nucleus
PMD: Premammillary nucleus, dorsal part
PMV: Premammillary nucleus, ventral part
PN: Paranigral nucleus
Pn: Pontine nucleus
PnC: Pontine reticular nucleus, caudal part
PnO: Pontine reticular nucleus, oral part
PoT: Posterior thalamic nuclear group, triangular part
PP: Peripeduncular nucleus
PPT: Posterior pretectal nucleus
PPTg: Pedunculopontine tegmental nucleus
Pr: Prepositus nucleus
PR: Prerubral field
Pr5DM: Principal sensory trigeminal nucleus, dorsomedial part
Pr5VL: Principal sensory trigeminal nucleus, ventrolateral part
PrL: Prelimbic cortex
PrS: Presubiculum
PSTh: Parasubthalamic nucleus
PT: Paratenial thalamic nucleus
PV: Paraventricular thalamic nucleus
PVA: Paraventricular thalamic nucleus, anterior part
PVP: Paraventricular thalamic nucleus, posterior part
Py: Pyramidal cell layer of the hippocampus
rCM: Central medial thalamic nucleus, rostral part
Re: Reuniens thalamic nucleus
Reth: Retroethmoid nucleus
Rh: Rhomboid thalamic nucleus
RI: Rostral interstitial nucleus of medial longitudinal fasciculus
RLi: Rostral linear nucleus of the raphe
RMC: Red nucleus, magnocellular part
RMg: Raphe magnus
Rob: Raphe obscurus
RPa: Raphe pallidus
RPC: Red nucleus, parvicellular part
RPF: Retroparafascicular nucleus
RPO: Rostral periolivary region
RR: Retrorubral area
RSA: Retrospenial agranular cortex
Rt: Reticular thalamic nucleus
RtTg: Reticulotegmental nucleus of the pons
RVL: Rostroventrolateral reticular nucleus
S: Subiculum
S1: Primary somatosensory cortex
S2: Secondary somatosensory cortex
SCh: Suprachiasmatic nucleus
scp: Superior cerebellar peduncle
SFi: Septofimbrial nucleus
SFO: Subfornical organ
SG: Suprageniculate thalamic nucleus
SGI: Superficial glial zone of the cochlear nuclei
Shi: Septohippocampal nucleus
SI: Substantia innominata
sm: Stria medullaris of the thalamus
SNC: Substantia nigra, compact part
SNL: Substantia nigra, lateral part
SNR: Substantia nigra, reticular part
SO: Supraoptic nucleus
Sol: Nucleus of the solitary tract
Sp5: Spinal trigeminal nucleus
SPFPC: Subparafascicular thalamic nucleus, parvicellular part
SPO: Superior paraolivary nucleus
SPTg: Subpeduncular tegmental nuclear
SpVe: Spinal vestibular nucleus
STh: Subthalamic nucleus
Su3: Supraoculomotor periaqueductal gray
Su3C: Supraoculomotor cap
Su5: Supratrigeminal nucleus
Sub: Submedius thalamic nucleus
SubB: Subbrachial nucleus
SuG: Superficial gray layer of the superior colliculus
SuML: Supramammillary nucleus, lateral part
SuMM: Supramammillary nucleus, medial part
SuVe: Superior vestibular nucleus
Te: Temporal cortex
TS: Triangular septal nucleus
Tu: Olfactory tubercle
tz: Trapezoid body
Tz: Nucleus of the trapezoid body
V1: Primary visual cortex
V2: Secondary visual cortex
VA: Ventral anterior thalamic nucleus
VCA: Ventral cochlear nucleus, anterior part
VCP: Ventral cochlear nucleus, posterior part
VDB: Nucleus of the vertical limb of the diagonal band
VeCb: Vestibulocerebellar nucleus
VL: Ventrolateral thalamic nucleus
VLGMC: Ventrolateral geniculate nucleus, magnocellular part
VLGPC: Ventrolateral geniculate nucleus, parvicellular part
VLL: Ventral nucleus of the lateral lemniscus
VLPAG: Ventrolateral periaqueductal gray
VM: Ventromedial thalamic nucleus
VMH: Ventromedial hypothalamic nucleus
VMPO: Ventromedial preoptic nucleus
VP: Ventral pallidum
VPL: Ventral posterolateral thalamic nucleus
VPM: Ventral posteromedial thalamic nucleus
VPPC (Gus): Gustatory thalamic nucleus
VRe: Ventral reuniens thalamic nucleus
VTg: Ventral tegmental nucleus
VTA: Ventral tegmental area
VTT: Ventral tenia tecta
X: Nucleus X
xcsp: Decussation of the superior cerebellar peduncle
Xi: Xiphoid thalamic nucleus
ZI: Zona incerta
