# Supplementary material for: A mu–delta opioid receptor brain atlas reveals neuronal co-occurrence in subcortical networks
Source: Brain Struct Funct. 2014 Mar 13;220(2):677–702. doi: 10.1007/s00429-014-0717-9 (PMC4341027; doi:10.1007/s00429-014-0717-9)

A mu-delta opioid receptor brain atlas reveals neuronal co-occurrence in subcortical networks.

Brain Structure & Function

Erbs E, Faget L, Scherrer G, Matifas A, Filliol D, Vonesch J-L, Koch M, Kessler P, Hentsch D, Birling M-C, Koutsourakis M, Vasseur L, Veinante P, Kieffer BL, Massotte D

Corresponding authors: Massotte D &Kieffer BL, IGBMC, massotte@igbmc.fr

Supplemental Figure 1: Molecular characterization of the MOR-mcherry knock-in mouse,
(A) Opioid receptor transcripts. Quantification of Oprm1 (mu) Oprd1 (delta), and Oprk1 (kappa) transcripts on mRNA preparations from Oprm1+/+ (black bars) or Oprm1mch/mch (grey bars) brains (n= 3 animals per genotype).

(B) Receptor signaling. [35S]GTPgS incorporation was measured on brain membranes from Oprm1+/+ (■),Oprm1+/mch (●) and Oprm1mch/mch (▲) mice following stimulation with the DOR selective agonist AR-M100390 or KOR selective agonist U50-488H. Data are the mean ± sem from independent experiments performed in triplicate (n=3 animals per genotype).

A

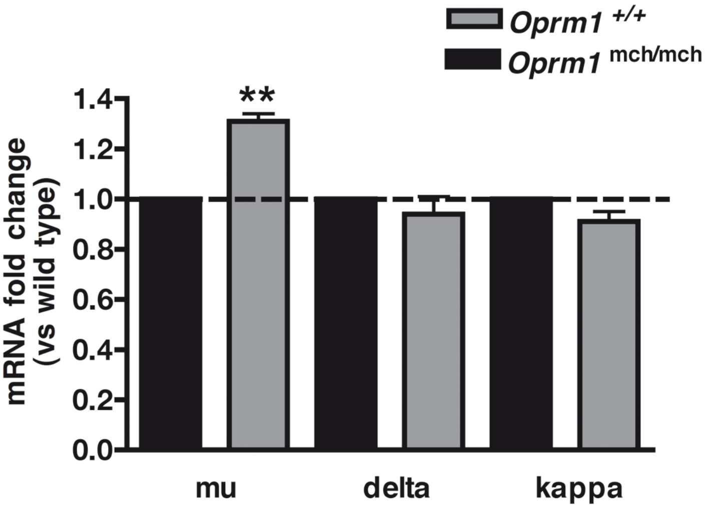

B

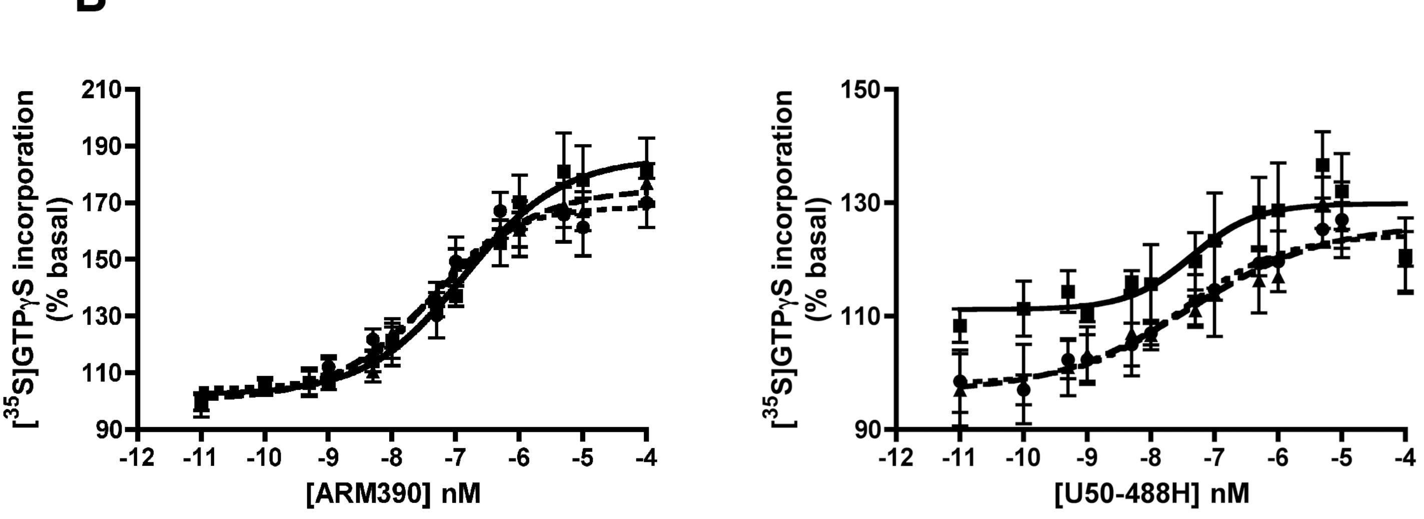

Supplement: Supplementary file 1 — Supplementary material 1 (PDF 632 kb) [file 429_2014_717_MOESM1_ESM.pdf]
